# Loss-of-Function Myeloperoxidase Mutations Are Associated with Increased Neutrophil Counts and Pustular Skin Disease

**DOI:** 10.1016/j.ajhg.2020.06.020

**Published:** 2020-08-05

**Authors:** Marta Vergnano, Maja Mockenhaupt, Natashia Benzian-Olsson, Maren Paulmann, Katarzyna Grys, Satveer K. Mahil, Charlotte Chaloner, Ines A. Barbosa, Suzannah August, A. David Burden, Siew-Eng Choon, Hywel Cooper, Alex A. Navarini, Nick J. Reynolds, Shyamal Wahie, Richard B. Warren, Andrew Wright, The APRICOT and PLUM study team, Thamir Abraham, Mahmud Ali, Suzannah August, David Baudry, Anthony Bewley, Hywel Cooper, John Ingram, Susan Kelly, Mohsen Korshid, Effie Ladoyanni, John McKenna, Freya Meynell, Richard Parslew, Prakash Patel, Angela Pushparajah, Nick Reynolds, Catherine Smith, Shyamal Wahie, Richard Warren, Andrew Wright, Ulrike Huffmeier, Patrick Baum, Sudha Visvanathan, Jonathan N. Barker, Catherine H. Smith, Francesca Capon

**Affiliations:** 1 Department of Medical and Molecular Genetics, School of Basic and Medical Biosciences, King’s College London, London SE1 9RT, UK; 2 St John’s Institute of Dermatology, School of Basic and Medical Biosciences, King's College London, London SE1 9RT, UK; 3 Department of Dermatology, Medical Centre-University of Freiburg, Freiburg 79106, Germany; 4 Poole Hospital NHS Foundation Trust, Poole BH15 2JB, UK; 5 Department of Dermatology, University of Glasgow, Glasgow G12 8QQ, UK; 6 Department of Dermatology, Sultanah Aminah Hospital, Clinical School Johor Bahru, Monash University, Malaysia; 7 Portsmouth Dermatology Centre, St Marys Hospital, Portsmouth PO3 6AD, UK; 8 Department of Dermatology & Allergy, University Hospital of Basel, Basel 4031, Switzerland; 9 Translational and Clinical Research Institute, Newcastle University, Newcastle upon Tyne NE2 4HH, UK and Department of Dermatology and NIHR Newcastle Biomedical Research Centre, Newcastle Hospitals NHS Foundation Trust, Newcastle upon Tyne NE2 4LP, UK; 10 Department of Dermatology, University Hospital of North Durham, Durham DH1 5TW, UK; 11 Dermatology Centre, Salford Royal NHS Foundation Trust, Manchester NIHR Biomedical Research Centre, University of Manchester, Manchester M6 8HD, UK; 12 Centre for Skin Sciences, St Lukes Hospital, Bradford BD5 0NA, UK; 13 Institute of Human Genetics, Friedrich-Alexander-Universität Erlangen-Nürnberg, Erlangen 91054, Germany; 14 Boehringer-Ingelheim International GmbH, Biberach 88397, Germany; 15 Boehringer-Ingelheim Pharmaceuticals, Ridgefield, CT 06877, USA

**Keywords:** *MPO*, myeloperoxidase, myeloperoxidase deficiency, neutrophils, generalized pustular psoriasis, GPP, acute generalized exanthematous pustulosis, AGEP

## Abstract

The identification of disease alleles underlying human autoinflammatory diseases can provide important insights into the mechanisms that maintain neutrophil homeostasis. Here, we focused our attention on generalized pustular psoriasis (GPP), a potentially life-threatening disorder presenting with cutaneous and systemic neutrophilia. Following the whole-exome sequencing of 19 unrelated affected individuals, we identified a subject harboring a homozygous splice-site mutation (c.2031−2A>C) in *MPO*. This encodes myeloperoxidase, an essential component of neutrophil azurophil granules. *MPO* screening in conditions phenotypically related to GPP uncovered further disease alleles in one subject with acral pustular psoriasis (c.2031−2A>C;c.2031−2A>C) and in two individuals with acute generalized exanthematous pustulosis (c.1705C>T;c.2031−2A>C and c.1552_1565del;c.1552_1565del). A subsequent analysis of UK Biobank data demonstrated that the c.2031−2A>C and c.1705C>T (p.Arg569Trp) disease alleles were also associated with increased neutrophil abundance in the general population (p = 5.1 × 10^−6^ and p = 3.6 × 10^−5^, respectively). The same applied to three further deleterious variants that had been genotyped in the cohort, with two alleles (c.995C>T [p.Ala332Val] and c.752T>C [p.Met251Thr]) yielding p values < 10^−10^. Finally, treatment of healthy neutrophils with an MPO inhibitor (4-Aminobenzoic acid hydrazide) increased cell viability and delayed apoptosis, highlighting a mechanism whereby *MPO* mutations affect granulocyte numbers. These findings identify *MPO* as a genetic determinant of pustular skin disease and neutrophil abundance. Given the recent interest in the development of MPO antagonists for the treatment of neurodegenerative disease, our results also suggest that the pro-inflammatory effects of these agents should be closely monitored.

## Main Text

A tight regulation of neutrophil numbers is crucial to innate immune homeostasis. As mature granulocytes do not divide, their accumulation depends on the balance between progenitor proliferation, release of differentiated cells into the bloodstream, and clearance of aging cells.^1^ Given the difficulty of manipulating primary neutrophils, the mechanisms that regulate these processes have mostly been investigated in animal models. In this context, the genetic characterization of human autoinflammatory diseases can provide crucial insights into the pathways that maintain neutrophil homeostasis.

Here we focused our attention on generalized pustular psoriasis (GPP [MIM: 614204]), a potentially life-threatening condition presenting with flares of neutrophilic skin inflammation (pustular eruptions), fever, increased production of acute phase reactants, and neutrophilia. While disease alleles have been described in *IL36RN*, *AP1S3*, and *CARD14*, the majority of affected individuals do not carry deleterious changes at these loci.^2^

**Figure 1:**
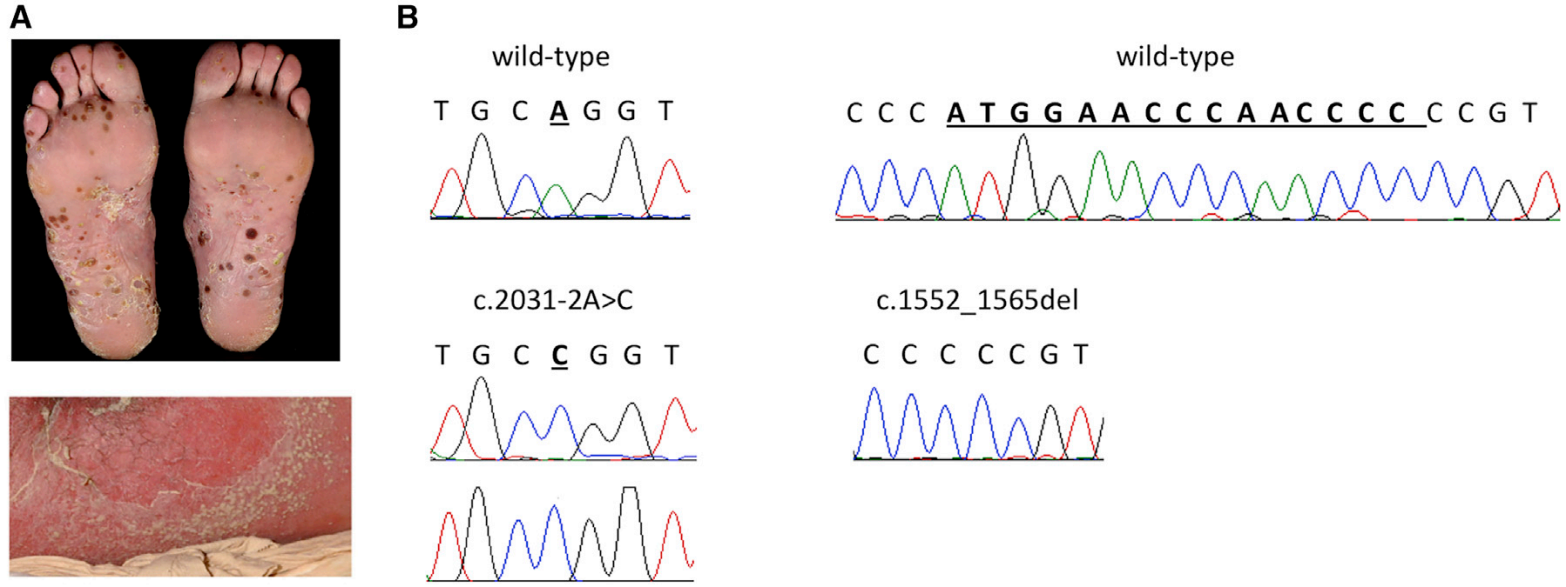
*MPO* Mutations Are Associated with Pustular Skin Disease (A) Typical presentation of generalized pustular psoriasis (bottom panel, showing skin pustulation on an erythematous background) and acral pustular psoriasis (top panel, showing neutrophil-filled pustules affecting the soles). (B) Validation of the disease alleles identified by whole-exome sequencing. The chromatograms show the c.2031−2A>C substitution observed in the GPP and APP subjects (left) and the c.1552_1565del deletion detected in a study participant with AGEP (right). The position of disease alleles is highlighted by bold, underlined font. Sanger sequencing could not be carried out in the c.2031−2A>C;c.1705C>T individual as no DNA was left for this subject.

To identify genetic determinants for GPP, we undertook whole-exome sequencing in 19 unrelated affected individuals of varying ancestry (Table S1, Figure 1A). Given the severity of the condition and the lack of parent-offspring transmissions, we hypothesized the presence of recessive loss-of-function alleles. We therefore filtered the variant profiles to retain rare homozygous changes predicted to cause premature protein truncation. This identified six candidate mutations, each affecting a single individual (Table S2).

The c.2031−2A>C substitution in *MPO* (MIM: 606989; GenBank: NM_000250) (Figure 1B) was selected for follow-up, as the gene encodes myeloperoxidase, a major component of neutrophil azurophilic granules. Of note, the c.2031−2A>C change has been previously observed in individuals presenting with myeloperoxidase deficiency (MPOD [MIM: 254600]) (Table S3), an inherited defect of neutrophil microbicidal activity.^3^ Specifically, Marchetti et al. demonstrated that the substitution affects splicing and leads to the production of a truncated protein lacking enzymatic activity.^3^

Here, the frequency of the c.2031−2C;c.2031−2C genotype among European GPP case subjects was much higher than that observed in the non-Finnish European exomes sequenced by the gnomAD consortium (5.3% versus 0.003%; p = 0.001) (Table 1). Given that frequency estimates obtained in small datasets are not always robust, the *MPO* coding region was next examined in a validation sample, including 14 GPP subjects and 109 individuals with acral variants of pustular psoriasis (APP). This uncovered a further study participant harboring a homozygous c.2031−2A>C substitution (Figure 1B, Table 2, and Supplemental Note).

**Table 1:** Frequency of the c.2031−2C;c.2031−2C Genotypes in Case Subjects versus Control Subjects

	**Genotype Counts (%)**		**p Value**
	**Cases**	**Controls**	
Discovery cohort	1/19 (5.3%)	2/56,7466 (0.003%)	0.001
Replication cohort	1/123 (0.8%)	0/32,264 (0%)	0.004
Combined study cohort	2/142 (1.4%)	2/89,010 (0.002%)	1.5 × 10^−5^

c.2031−2A>C was the only truncating change observed in the homozygous state in the control subjects, obviating the need for a burden association test.

**Table 2:** Disease Features Observed in Individuals with Bi-allelic *MPO* Mutations

**Subjects**	**Sex**	**Age of Onset**	**Diagnosis**^a^	**Systemic Involvement**	**Genotype**
GYFAP0014	F	36	GPP	fever and neutrophilia	c.2031−2A>C;c.2031−2A>C
DDPLM0001	F	24	APP	–	c.2031−2A>C;c.2031−2A>C
SCAR2124	F	80	AGEP (methotrexate)	fever and neutrophilia	c.1552_1565del;c.1552_1565del
SCAR2567	F	69	AGEP (hydroxychloroquine)	fever	c.2031−2A>C;c.1705C>T

None of the affected individuals reported a history of recurrent infections.

a The most likely culprit drug for each AGEP subject is reported in parentheses. AGEP, acute generalized exanthematous pustulosis; APP, acral pustular psoriasis; GPP, generalized pustular psoriasis.

While no other bi-allelic or truncating *MPO* changes were detected (Table S4), a comparison of the replication cohort against a second gnomAD dataset (32,264 Non-Finnish European genomes) confirmed the elevated frequency of the c.2031−2C;c.2031−2C genotype in case subjects versus control subjects (0.8% versus 0%; p = 0.004) (Table 1). Finally, the analysis of the combined study resource (142 case subjects versus 89,010 control subjects) yielded a p value of 1.5 × 10^−5^ (Table 1). Importantly, the c.2031−2C;c.2031−2C genotype was also absent from 590 British exomes processed with our in-house pipeline. Thus, the association with GPP/-APP is unlikely to be a technical artifact or to reflect population stratification between case and control subjects.

To further investigate the impact of *MPO* recessive alleles, we queried exome-sequencing data available for 96 unrelated individuals affected by acute generalized exanthematous pustulosis (AGEP). This is a severe cutaneous adverse reaction, which can be triggered by drugs (mostly antibiotics and antifungals), leading to flares of skin pustulation, fever, and systemic neutrophilia.^4^ Our analysis identified an affected individual, who was homozygous for a 14 bp *MPO* deletion (c.1552_1565del [p.Met519Profs^∗^21]). A second study participant had inherited the c.2031−2A>C substitution previously observed in GPP/APP, in conjunction with a damaging c.1705C>T (p.Arg569Trp) change (CADD score: 35.0) (Table 2, Supplemental Note).

Of note, homozygous c.1705C>T (p.Arg569Trp) mutations have been repeatedly observed in individuals affected by MPOD.^5^^,^^6^ A c.1555_1568del variant, which overlaps the c.1552_1565del variant described here, has also been documented in an affected-relative pair, where it triggered nonsense-mediated decay in at least one individual^7^ (Table S3). Thus, all the *MPO* alleles observed in our dataset have a well-established impact on protein function.

MPOD is a mild immune deficiency that is clinically well characterized. We therefore undertook a systematic literature review, to better understand the connection between *MPO* mutations, MPOD, and skin pustulation. We examined 28 articles describing the presentation of MPOD in 217 individuals. This uncovered four case reports where the disease manifested with pustular eruptions and a fifth where it was associated with the severe neutrophilic dermatosis known as pyoderma gangrenosum (Table S5). Given the very low prevalence of the above conditions (<1:100,000), these observations strengthen the link between MPO dysfunction and neutrophilic inflammation.

**Figure 2:**
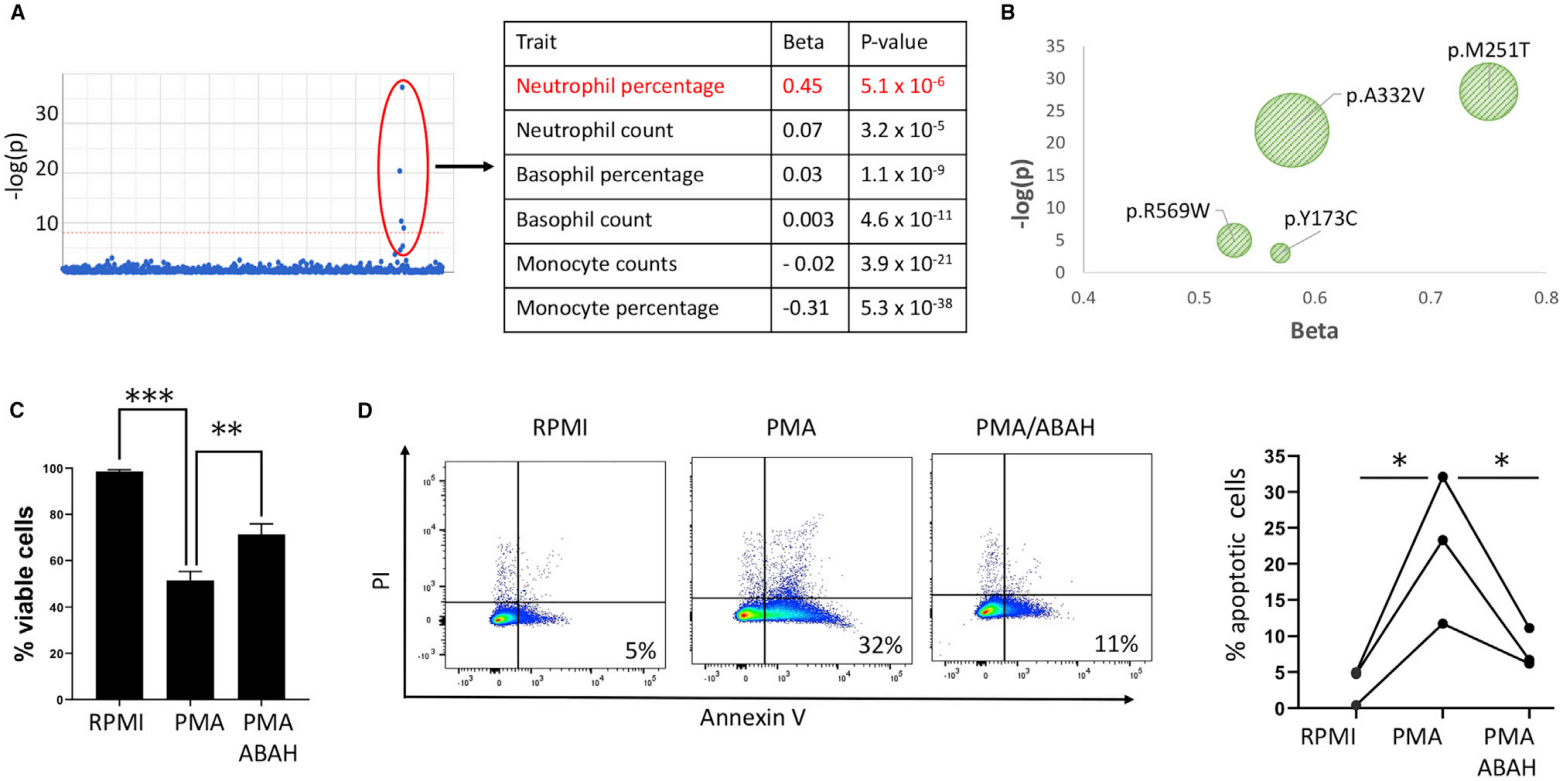
*MPO* Mutations Are Associated with Increased Neutrophil Counts and Delayed Apoptosis (A) Manhattan plot where each dot represents the association between c.2031−2A and a clinical trait. The p values for phenotypes related to leukocyte counts are highlighted with a red circle and reported on the right, alongside the effect sizes (beta). (B) Association between MPO deficiency alleles and neutrophil percentage. The size of each bubble represents the frequency of the mutation in UK Biobank. (C and D) ABAH pre-treatment of cells stimulated with PMA increases viability (C) and downregulates apoptosis (D). In (C) data are presented as the mean (±SD) of four experiments carried out in triplicate. In (D) each line represents an independent healthy donor. A representative set of flow cytometry plots is shown on the left with the percentage of apoptotic cells (AnnexinV^+^, PI^-^ population) for each condition. RPMI = untreated (medium only); ^∗^p < 0.05 ^∗∗^p < 0.01; ^∗∗∗^p < 0.001 (non-parametric ANOVA).

To investigate the mechanisms whereby *MPO* mutations contribute to disease, we explored the phenotypic effects of the c.2031−2A>C variant though a Phenome-Wide Association study (PheWAS). We queried the UK Biobank dataset, which includes genotype information and health data for a well-characterized population cohort (>450,000 individuals).^8^ While the fraction of c.2031−2C homozygotes (0.003%) (Table S3) present in the biobank was very small, c.2031−2A>C heterozygotes accounted for approximately 1% of study participants. Thus, we were able to analyze the c.2031−2A>C genotypes against 778 available phenotypes. This revealed that the traits showing the most significant associations with c.2031−2A>C were related to leukocyte counts. In this context, the largest effect size (beta) was observed for the association with neutrophil abundance (beta = 0.45; p = 5.1 × 10^−6^; Figure 2A). To validate these findings, we examined the c.1705C>T (p.Arg569Trp) change and three additional MPOD alleles (c.518A>G [p.Tyr173Cys], c.752T>C [p.Met251Thr], c.995C>T [p.Ala332Val]) for which genotype data were available in UK Biobank (Table S3). We found that all were associated with increased neutrophil accumulation, with p values ranging from 0.008 to 3.9 × 10^−28^ (Figure 2B).

To explore the pathways underlying the effects of *MPO* alleles on granulocyte numbers, we examined RNA-sequencing profiles generated in pure neutrophil populations (see Supplemental Subjects and Methods). Specifically, we compared gene expression in the c.2031−2A>C homozygous GPP individual versus 11 healthy control subjects. We observed that *MPO* was expressed at comparable levels in the affected subject and the unaffected control subjects (FDR > 0.5). As c.2031−2A>C affects the splicing of the last exon, this is in keeping with the expectation of an escape from nonsense-mediated decay.

Conversely, we found that 95 genes were upregulated in the affected individual (FDR < 0.05) (Table S6). The majority of these loci (85/95) were not overexpressed in 7 unrelated individuals with GPP (all *MPO* wild type) examined in parallel, indicating that the changes are unlikely to be a secondary effect of inflammation.

While the experiment was limited by the modest sample size and the number of differentially expressed genes was too small for pathway enrichment analyses, we noted that two of the five most upregulated loci (*PBK* and *GUCYA2*) encode proteins (PDZ binding kinase and soluble guanylate cyclase alpha-2 subunit) that can inhibit apoptosis.^9^^,^^10^ This suggests that *MPO* mutations may affect neutrophil survival.

We investigated this possibility by using a myeloperoxidase inhibitor (4-Aminobenzoic acid hydrazide, ABAH) to mimic the effects of *MPO* disease alleles in cell culture experiments. We induced neutrophil apoptosis through Phorbol 12-myristate 13-acetate (PMA) stimulation and assessed the effects of ABAH pre-treatment on this process. While PMA caused substantial neutrophil death, we found that ABAH supplementation caused an increase in cell viability (Figure 2C) and a reduction in the number of apoptotic cells (Figure 2D). Thus, neutrophil apoptosis is downregulated in the absence of MPO activity.

Our findings (and those independently reported by Haskamp et al.^11^) demonstrate a significant association between *MPO* mutations and pustular skin disease. While the disease alleles described here have also been implicated in MPOD, we did not observe any evidence of immune deficiency in the individuals examined in this study. Likewise, pustular skin disease is present in only a fraction of people affected by MPOD. Thus, it is tempting to speculate that the manifestations of *MPO* mutations may be influenced by background polygenic variation, especially as a similar phenomenon has been documented in rare hematological phenotypes.^12^ Of note, the c.2031−2C;c.2031−2C GPP individual described here also harbored a deleterious *AP1S3* allele (GenBank: NM_001039569.2; c.97C>T [p.Arg33Trp]),^13^ which further supports the involvement of genetic modifiers.

An increased prevalence of spondyloarthropathy has also been reported among individuals with MPO deficiency.^14^ This suggests that the disruption of neutrophil apoptosis may affect immune homeostasis in multiple organs. Given that MPO inhibitors are being developed for the treatment of neurodegenerative disease,^15^ our data suggest that the inflammatory side effects of these agents should be closely monitored during clinical trials.

The results of our PheWAS indicate that the effects of *MPO* alleles are likely to be mediated by a systemic upregulation of neutrophil numbers. Of note, a significant association between granulocyte abundance and a common *MPO* variant has previously been documented,^16^ further supporting the role of the gene in neutrophil homeostasis.

Further studies will be required to dissect the molecular mechanisms whereby MPO deficiency downregulates cell death. Given that *PBK* (one of the most upregulated genes in the c.2031−2A>C homozygous individual) is an inhibitor of myeloid cell apoptosis,^9^ its role is worthy of further examination. A proposed link between MPO-related oxidative stress, NF-κB activation, and apoptotic signaling^17^ should also be investigated. While experimentally demanding, these studies have the potential to illuminate key regulators of innate immune homeostasis and uncover new candidate genes for neutrophilic conditions.

## Data and Code Availability

The neutrophil RNA-sequencing dataset described in this study may be obtained from the Gene Expression Omnibus using identifier GSE123787.

## Declaration of Interests

A.D.B. has received funding from Boehringer-Ingelheim. C.H.S. has been a principal (or co-) investigator on commercially sponsored clinical trials and investigator-led studies funded by AbbVie, GaxoSmithKline, Janssen, Novartis, Pfizer, Regeneron, Roche, Sanquin, Celgene, Sanofi, LEO Pharma, Boehringer Ingleheim, and UCB Pharma. F.C. has received funding from Boehringer-Ingelheim and consultancy fees from AnaptysBio. J.N.B. has received funding and fees from Abbvie, Boehringer-Ingelheim, Bristol Myers Squibb, Celegene, Ely Lily, Novartis, Pfizer, Samsung, Sienna, and Sun Pharma. N.J.R. has received research funding for clinical trials from AnaptysBio through Newcastle Hospitals NHS Foundation Trust. P.B. and S.V. are Boehringer-Ingelheim employees. S.W. has received non-financial support (sponsorship to attend dermatology conferences) from Janssen, Abbvie, Novartis, and Almirall.

## Web Resources

CADD, https://cadd.gs.washington.edu/
ClinVar, https://www.ncbi.nlm.nih.gov/clinvar/
EGA, https://www.ebi.ac.uk/ega/home
GeneAtlas, http://geneatlas.roslin.ed.ac.uk/
Gene Expression Omnibus, https://www.ncbi.nlm.nih.gov/gds
gnomAD server, https://gnomad.broadinstitute.org/
OMIM, https://omim.org/
PubMed, https://www.ncbi.nlm.nih.gov/pubmed/

## Supplemental Data

Document S1. Supplemental Note, Supplemental Subjects and Methods, and Tables S1–S7

Document S2. Article plus Supplemental Information
