# Loss-of-function myeloperoxidase mutations are associated with increased neutrophil counts and pustular skin disease

**DOI:** 10.1016/j.ajhg.2021.03.001

**Published:** 2021-04-01

**Authors:** Marta Vergnano, Maja Mockenhaupt, Natashia Benzian-Olsson, Maren Paulmann, Katarzyna Grys, Satveer K. Mahil, Charlotte Chaloner, Ines A. Barbosa, Suzannah August, A. David Burden, Siew-Eng Choon, Hywel Cooper, Alex A. Navarini, Nick J. Reynolds, Shyamal Wahie, Richard B. Warren, Andrew Wright, The APRICOT and PLUM study team, Thamir Abraham, Mahmud Ali, Suzannah August, David Baudry, Anthony Bewley, Hywel Cooper, Christopher E.M. Griffiths, John Ingram, Susan Kelly, Mohsen Korshid, Effie Ladoyanni, John McKenna, Freya Meynell, Richard Parslew, Prakash Patel, Angela Pushparajah, Nick Reynolds, Catherine Smith, Shyamal Wahie, Richard Warren, Andrew Wright, Ulrike Huffmeier, Patrick Baum, Sudha Visvanathan, Jonathan N. Barker, Catherine H. Smith, Francesca Capon

(The American Journal of Human Genetics *107*, 539–543; September 3, 2020)

In the version of this paper originally published, Christopher E.M. Griffith was accidently omitted from the list of members of The APRICOT and PLUM study team. This has been corrected online. The authors apologize for this error.

