# Supplementary material for: Loss-of-Function Myeloperoxidase Mutations Are Associated with Increased Neutrophil Counts and Pustular Skin Disease
Source: Am J Hum Genet. 2020 Aug 5;107(3):539–43. doi: 10.1016/j.ajhg.2020.06.020 (PMC7477255; doi:10.1016/j.ajhg.2020.06.020)
Supplement: Document S1. Supplemental Note, Supplemental Subjects and Methods, and Tables S1–S7 [file mmc1.pdf]

The American Journal of Human Genetics, Volume *107*

Supplemental Data

Loss-of-Function Myeloperoxidase Mutations Are Associated with Increased Neutrophil Counts and Pustular Skin Disease

Marta Vergnano, Maja Mockenhaupt, Natashia Benzian-Olsson, Maren Paulmann, Katarzyna Grys, Satveer K. Mahil, Charlotte Chaloner, Ines A. Barbosa, Suzannah August, A. David Burden, Siew-Eng Choon, Hywel Cooper, Alex A. Navarini, Nick J. Reynolds, Shyamal Wahie, Richard B. Warren, Andrew Wright, The APRICOT and PLUM study team, Ulrike Huffmeier, Patrick Baum, Sudha Visvanathan, Jonathan N. Barker, Catherine H. Smith, and Francesca Capon

Supplemental note: Membership of the PLUM and APRICOT study team

The following members of the PLUM and APRICOT study team contributed to this work:

Thamir Abraham (Peterborough city Hospital),Mahmud Ali (Worthing Hospital), Suzannah August (Poole Hospital), David Baudry (Guy's Hospital, London), Anthony Bewley (Whipps Cross University Hospital, London), Hywel Cooper (St Marys Hospital, Portsmouth), John Ingram (University Hospital of Wales), Susan Kelly (The Royal Shrewsbury Hospital), Mohsen Korshid (Basildon Hospital), Effie Ladoyanni (Russell's Hall Hospital, Dudley), John McKenna (Leicester Royal Infirmary),Freya Meynell (Guy's Hospital, London), Richard Parslew (Broadgreen Hospital, Liverpool), Prakash Patel (Guy's Hospital, London), Angela Pushparajah (Guy's Hospital, London), Nick Reynolds (Newcastle Hospitals), Catherine Smith (Guy's Hospital, London), Shyamal Wahie (University Hospital of North Durham and Darlington Memorial Hospital), Richard Warren (Salford Royal Infirmary), Andrew Wright (St Lukes Hospital, Bradford).

Additional affected individuals were recruited by A David Burden (Glasgow Western Infirmary), Siew-Eng Choon (Johor Bahru Hospital, Malaysia), Brian Kirby (St Vincent University Hospital, Dublin, Ireland), Alexander Navarini (University Hospital Zurich, Switzerland) and Marieke Sieger (Radboud Medical Centre, Nijmegen, The Netherlands).

Supplemental Note: Case Reports

The four individuals harbouring bi-allelic *MPO* mutations are described below.

GYFAP0014 is a British 54-year-old female of European descent. She was diagnosed with Acrodermatitis Continua of Hallopeau (ACH, a localised pustular eruption affecting the nails and distal phalanxes) aged 10. She also has a history of generalised pustular psoriasis that flared during pregnancy (at age 36 and 39) with widespread pustulation, high-fever, severe neutrophilia (>15,000 cells/mm^3) and elevated CRP levels (>100mg/L). Following several years of quiescent disease on infliximab infusions (5mg per kg every 10 weeks) and oral methotrexate (5mg per week) co-therapy, both treatments were withdrawn at age 52. Skin clearance has subsequently been maintained (2 years follow up to date). Her comorbidities include anxiety and depression. She has a body mass index (BMI) of 30. She does not have concurrent plaque psoriasis or psoriatic arthritis, and there is no family history of psoriasis.

DDPLM001 is a 35-year-old British female of European descent. She has been suffering from palmoplantar pustulosis since she was 24. She also has ACH with nail loss on her left hand and is affected by chronic plaque psoriasis and psoriatic arthritis. Her BMI is 26 and she is an ex-smoker. She has been receiving ciclosporin for the past 3 years with good effect. Her previous treatments include methotrexate and oral PUVA. There is no family history of psoriasis.

SCAR2124 is a female from Germany who presented with acute generalised exanthematous pustulosis (AGEP) at age 80, requiring an admission to hospital for 23 days. She suffered from widespread pustulation, fever and severe neutrophilia (>15,000 cells/mm^3). The culprit drugs (in order of suspicion) are methotrexate, pantoprazole and sultamicillin (ampicillin/sulbactam).

SCAR2567 is a female from Germany who presented with AGEP secondary to hydroxychloroquine at age 70. She suffered from high fever and pustulation, requiring an admission to hospital for 5 days. Her comorbidities include previous giant cell arteritis.

Supplemental Subjects and Methods

Study participants

The study was carried out according to the principles of the declaration of Helsinki and was approved by the ethics committees of participating institutions. Written informed consent was also obtained from all participants. Ninety-four individuals with generalised or acral forms of pustular psoriasis were ascertained through the APRICOT clinical trial (Anakinra in Pustular psoriasis, Response In a Controlled Trial; EudraCT n. 2015-003600-23) and its sister mechanistic study PLUM (Pustular psoriasis, eLucidating Underlying Mechanisms). Forty-eight additional cases were recruited outside of these programmes at St John's Institute of Dermatology (London, UK), Glasgow Western Infirmary (UK), Zurich University Hospital (Switzerland) and Hospital Sultanah Aminah (Johor Bahru, Malaysia). Pustular psoriasis was diagnosed by expert dermatologists, based on clinical examination and/or published consensus criteria[1]. Given various reports of digenic inheritance in GPP[2], individuals harbouring deleterious alleles at known disease loci were not excluded from the study cohort.

Subjects affected by AGEP were actively recruited by a network of hospitals in six countries. All were interviewed by trained investigators who recorded detailed information on the clinical course of the disease, previous medical history and suspected causative factors including infections and medications. Drug intake in the month before hospital admission was also recorded in a systematic way. All case notes were reviewed by a multinational expert committee of dermatologists blinded for information on risk factors. Clinical photographs were viewed together with clinical information from the case record forms and histological information. Cases were then assessed with a published scoring system[3] and were either excluded from the cohort or classified as definite, probable or possible. Only definite and probable cases were included in this study.

Given previous evidence of digenic inheritance in pustular skin disorders, affected individuals were included in the study, even if they harboured mutations at known disease loci[2, 4].

Healthy volunteers for MPO inhibition studies were recruited among the personnel of St John's Institute of Dermatology.

Whole-exome sequencing and Sanger sequencing

Five affected individuals were exome sequenced as part of previously published studies[4, 5]. For the remaining samples libraries were prepared with the Agilent SureSelect Human All Exome kit and run on an Illumina HiSeq3000 instrument. Paired-end reads were aligned to the hg19 reference genome with Novoalign (Novocraft Technologies) After the removal of duplicate reads, SNPs and small insertion deletions were identified with SAMtools[6]. Finally, variant files were annotated with ANNOVAR[7]. The same pipeline was used to process 590 control exomes generated by the UK Institute of Cancer Research[8].

Given that none of the affected individuals had an affected parent, exome profiles were filtered based on an autosomal recessive mode of inheritance. To maximise the likelihood of detecting deleterious loss-of-function alleles, variants were only retained if they: i) caused splicing, stop-gain, stop-loss and frameshift changes; ii) occurred in homozygosis; iii) had a MAF ≤ 0.01 in the ExAC dataset and in our in-house sequencing database; iv) were associated with CADD pathogenicity scores>15[9].

Upon completion of the filtering process, the *MPO* coding region was screened by Sanger sequencing in 14 additional GPP subjects, using the primers listed in Table S7. The gene was also examined in 109 APP sufferers, by interrogating whole-exome data generated as described above.

Systematic literature review

The PubMed database was queried using the terms (MPO deficiency) or (myeloperoxidase deficiency). The search, which was restricted to articles written in English and published before June 2019, retrieved 225 papers. Following the removal of reviews and irrelevant studies (e.g. characterisation of animal models and cell lines), 28 original articles were examined in detail.

Neutrophil RNA-sequencing

Neutrophil RNA-sequencing of 8 unrelated GPP individuals and 11 healthy controls was carried out as part of a previous study[10]. Briefly, the MACSxpress Whole Blood Neutrophil Isolation Kit (Miltenyi Biotec) was used to isolate high-purity (>95%) neutrophil populations. Following mRNA capture, samples were sequenced on a NextSeq 500 Illumina platform. Reads were then aligned to the HG38 genome and quantified with HTseq-count[10].

Here, the expression profile of the individual carrying the c.2031-2A>C variant was compared to that of the 11 controls. Fold changes (FC) were calculated by dividing the gene expression levels (RPKM) observed in the proband by the mean RPKM of the controls. FC values were then converted into z-scores by normalising their distribution to a mean of zero and standard deviation of 1. Finally, p-values were computed based on the normal distribution and adjusted for multiple testing (Bonferroni correction). The specificity of the gene expression changes was assessed through a parallel analysis of the 7 remaining GPP subjects, which were compared to the 11 healthy controls.

Cell viability and apoptosis assays

Neutrophils were purified using the MACSxpress Whole Blood Neutrophil Isolation Kit (Miltenyi) and 10^6 cells were cultured in 500µl RPMI (Gibco) supplemented with 1% BSA (Sigma-Aldrich). For viability assays, cultures were treated with 400µM ABAH (Abcam) for 4h after which 100nM PMA (Cayman Chemicals) or vehicle was added to the medium. After 20h, live cells were counted with a Nucleocounter NC-200 (ChemMetec). For apoptosis assays, cultures were treated with 400µM ABAH for 4h after which 25nM PMA or vehicle was added to the medium. After 20h, cells were processed

with the Annexin-V-FLUOS Staining Kit (Sigma Aldrich) and analysed on a BD FACSCanto™ II Flow Cytometry System. All experiments were carried out three times.

Analysis of UK Biobank dataset

The phenotypes associated with the c.2031-2A>C mutation were identified with the PheWAS function of The Gene ATLAS browser (a database reporting the results of 778 genome-wide association studies, carried out in UK Biobank using an additive genetic model[11]). To validate the association between c.2031-2A>C and neutrophil count variation, the details of additional MPOD alleles were retrieved from the OMIM database and used in further Gene ATLAS queries.

Statistical tests

The frequency of the c.2031-2A>C/c.2031-2A>C genotype was compared in cases vs. gnomAD controls[12] (non-Finnish European exomes v.2.1.1 and non-Finnish European genomes v.3), using Fisher's exact test. The effects of ABAH on PMA treated cells were assessed with a non-parametric ANOVA (Friedman's test).

Supplemental Tables

Table S1: Composition of the study cohort

Diagnosis	Sex	Ancestry	Average age of onset
GPP-WES (n=19)	4 males<br>15 females	European (n=9); Asian (n=9); Romani (n=1)	31 years
GPP-Sanger (n=14)	3 males<br>11 females	European (n=10); Asian (n=4)	40 years
APP (n=109)	22 males<br>87 females	European (n=109)	44 years

APP, Acral Pustular psoriasis; GPP, Generalized Pustular Psoriasis; WES, whole-exome sequencing

Table S2: Rare loss-of-function variants identified in 19 unrelated GPP individuals

Gene	Variant	Global MAF	Pathogenicity score (CADD)[1]	Gene function
ARAP1	c.4070+5G>T	0.0005	22.8	Modulates actin cytoskeleton remodelling
CCSER2	c.1868+5T>A	0.0003	15.8	Might play a role in microtubule bundling
DMBT1	c.1504C>T	0.00002	35.0	Candidate tumour suppressor gene for brain, lung, oesophageal, gastric, and colorectal cancers
EIF4G1	c.698-3C>T	0.0016	16.5	Encodes a component of the EIF4F protein complex
FAM83A	c.871C>T	0.0018	36.0	Probable proto-oncogene activating AKT/TOR signalling
MPO	**c.2031-2A>C**	**0.0043**	**32.0**	**Major component of azurophilic granules; produces hypohalous acids that are essential to the microbicidal activity of neutrophils**

[1]CADD scores>15.0 are considered as evidence of pathogenicity; MAF, minor allele frequency

Table S3: Myeloperoxidase deficiency alleles reported in the OMIM database

cDNA (protein) change	Clinvar accession n.	dbSNP ID	UK Biobank MAF (n. of homozygotes; %)
c.2031-2A>C (p.Phe678_Ser745delins(71))	VCV000003632	rs35897051	0.0064 (14; 0.003%)
c.1715T>G (p.Leu572Trp)	VCV000003631	rs119469012	n/a
c.1705C>T (p.Arg569Trp)	VCV000003626	rs119468010	0.0035 (4; 0.001%)
c.1555_1568del (p.Met519fs)	VCV000003629	rs536522394	n/a
c.1501G>A (p.Gly501Ser)	VCV000003634	rs119469013	n/a
c.1495C>T (p.Arg499Cys)	VCV000003635	rs119469014	n/a
c.995C>T (p.Ala332Val)	VCV000003630	rs28730837	0.016 (121; 0.03%)
c.752T>C (p.Met251Thr)	VCV000003628	rs56378716	0.013 (76; 0.02%)
c.518A>G (p.Tyr173Cys)	VCV000003627	rs78950939	0.0012 (1; 0.0002%)

MAF, minor allele frequency; n/a: not available

Table S4: Rare and low-frequency *MPO* variants observed in the replication cohort[1]

cDNA change	Protein change	dbSNP ID	MAF among gnomAD controls	MAF among cases	Mutation class (status)
c.752T>C	p.Met251Thr	rs56378716	0.014	0.012	missense (heterozygous)
c.995C>T	p.Ala332Val	rs28730837	0.018	0.016	missense (heterozygous)
c.1379G>A	p.Arg460Gln	rs149133270	0.002	0.004	missense (heterozygous)
c.1643G>A	p.Arg548Gln	rs144371238	0.0001	0.004	missense (heterozygous)
c.1705C>T	p.Arg569Trp	rs119468010	0.003	0.004	missense (heterozygous)
c.2149A>G	p.Ile717Val	rs2759	0.029	0.032	missense (heterozygous)

[1]Excluding the c.2031-2A>C allele, which is described in the main text

Table S5: Association of pustular skin disease and myeloperoxidase deficiency

Case report	*Reference*
• 46-year-old male with MPOD, plaque psoriasis and GPP.<br>• Identical twin with mild pustular psoriasis.	Stendahl and Lindgren[13]
• 61-year-old female with MPOD and annular pustular psoriasis	De Argila et al.[14]
• 20-year-old male with MPOD developed generalized pustular eruptions following injury	Nguyen and Katner[15]
• 81-year-old female with MPOD and pyoderma gangrenosum	Disdier et al.[16]

GPP, Generalized pustular psoriasis; MPOD, myeloperoxidase deficiency

Table S6: Genes upregulated in the neutrophils of the c.2031-2A>C homozygous GPP individual

Gene	fold change	z-score	p value	adj p value
PBK	33.00	27.02	1.06E-159	1.66E-155
GPR33	33.00	27.02	1.06E-159	1.66E-155
COL14A1	27.50	22.37	7.88E-110	1.24E-105
GUCY1A2	22.00	17.72	2.40E-69	3.77E-65
SLC10A4	22.00	17.72	2.40E-69	3.77E-65
DEFB1	22.00	17.72	2.40E-69	3.77E-65
DPP10	22.00	17.72	2.40E-69	3.77E-65
PURG	22.00	17.72	2.40E-69	3.77E-65
CALCB	22.00	17.72	2.40E-69	3.77E-65
DUOXA1	22.00	17.72	2.40E-69	3.77E-65
BDKRB2	22.00	17.72	2.40E-69	3.77E-65
DEFA3	21.56	17.35	1.65E-66	2.58E-62
METTL7B	15.40	12.15	3.71E-33	5.82E-29
NNMT	15.40	12.15	3.71E-33	5.82E-29
SLC22A16	13.17	10.26	5.67E-24	8.89E-20
CES1	13.02	10.13	2.07E-23	3.24E-19
USP2	11.00	8.43	1.53E-16	2.40E-12
C11orf45	11.00	8.43	1.53E-16	2.40E-12
CLEC3B	11.00	8.43	1.53E-16	2.40E-12
MAGEL2	11.00	8.43	1.53E-16	2.40E-12
FRAS1	11.00	8.43	1.53E-16	2.40E-12
MUC12	11.00	8.43	1.53E-16	2.40E-12
SLIT3	11.00	8.43	1.53E-16	2.40E-12
ALPK2	11.00	8.43	1.53E-16	2.40E-12
HCN4	11.00	8.43	1.53E-16	2.40E-12
IGFBP5	11.00	8.43	1.53E-16	2.40E-12
C9orf47	11.00	8.43	1.53E-16	2.40E-12
GLRA3	11.00	8.43	1.53E-16	2.40E-12
TERT	11.00	8.43	1.53E-16	2.40E-12
CLDN1	11.00	8.43	1.53E-16	2.40E-12
PLEKHS1	11.00	8.43	1.53E-16	2.40E-12
MISP	11.00	8.43	1.53E-16	2.40E-12
KSR2	11.00	8.43	1.53E-16	2.40E-12
PRSS16	11.00	8.43	1.53E-16	2.40E-12
LRRC66	11.00	8.43	1.53E-16	2.40E-12
SLC26A7	11.00	8.43	1.53E-16	2.40E-12
MUSK	11.00	8.43	1.53E-16	2.40E-12
PDYN	11.00	8.43	1.53E-16	2.40E-12
SCG2	11.00	8.43	1.53E-16	2.40E-12
MEPE	11.00	8.43	1.53E-16	2.40E-12
SLC7A13	11.00	8.43	1.53E-16	2.40E-12
ZNF474	11.00	8.43	1.53E-16	2.40E-12
SHC2	11.00	8.43	1.53E-16	2.40E-12
SUN3	11.00	8.43	1.53E-16	2.40E-12
EN2	11.00	8.43	1.53E-16	2.40E-12
ATP4B	11.00	8.43	1.53E-16	2.40E-12

BMP4	11.00	8.43	1.53E-16	2.40E-12
RASGEF1C	11.00	8.43	1.53E-16	2.40E-12
ACTL6B	11.00	8.43	1.53E-16	2.40E-12
SLC22A3	11.00	8.43	1.53E-16	2.40E-12
KLRC2	11.00	8.43	1.53E-16	2.40E-12
CXCL13	11.00	8.43	1.53E-16	2.40E-12
VCX	11.00	8.43	1.53E-16	2.40E-12
OR5K2	11.00	8.43	1.53E-16	2.40E-12
KITLG	11.00	8.43	1.53E-16	2.40E-12
TTC36	11.00	8.43	1.53E-16	2.40E-12
IFNB1	11.00	8.43	1.53E-16	2.40E-12
DPPA5	11.00	8.43	1.53E-16	2.40E-12
C6orf99	11.00	8.43	1.53E-16	2.40E-12
SLC10A6	11.00	8.43	1.53E-16	2.40E-12
LHB	11.00	8.43	1.53E-16	2.40E-12
CXCL11	11.00	8.43	1.53E-16	2.40E-12
WFDC2	11.00	8.43	1.53E-16	2.40E-12
CAPN11	11.00	8.43	1.53E-16	2.40E-12
XAGE3	11.00	8.43	1.53E-16	2.40E-12
MLN	11.00	8.43	1.53E-16	2.40E-12
IGFL3	11.00	8.43	1.53E-16	2.40E-12
SPINK1	11.00	8.43	1.53E-16	2.40E-12
HIST1H2BM	11.00	8.43	1.53E-16	2.40E-12
SLC22A31	11.00	8.43	1.53E-16	2.40E-12
DIRAS2	11.00	8.43	1.53E-16	2.40E-12
PGA3	11.00	8.43	1.53E-16	2.40E-12
FIGNL2	11.00	8.43	1.53E-16	2.40E-12
BCAR1	9.26	6.96	1.23E-11	1.93E-07
CRYGD	9.00	6.74	5.63E-11	8.83E-07
AZU1	8.96	6.70	7.17E-11	1.12E-06
NRP1	8.80	6.57	1.73E-10	2.72E-06
ELANE	8.52	6.33	7.98E-10	1.25E-05
PXMP2	8.25	6.10	3.30E-09	5.17E-05
LURAP1L	8.25	6.10	3.30E-09	5.17E-05
ABCA8	7.86	5.77	2.37E-08	3.71E-04
TPSD1	7.86	5.77	2.37E-08	3.71E-04
EDA2R	7.86	5.77	2.37E-08	3.71E-04
CABP1	7.86	5.77	2.37E-08	3.71E-04
GPR15	7.75	5.68	3.97E-08	6.23E-04
BUB1B	7.40	5.39	2.01E-07	3.14E-03
PTCRA	7.33	5.33	2.76E-07	4.33E-03
C2orf80	7.33	5.33	2.76E-07	4.33E-03
MARCH2	7.33	5.33	2.76E-07	4.33E-03
NDNF	7.33	5.33	2.76E-07	4.33E-03
STEAP1B	7.33	5.33	2.76E-07	4.33E-03
OR52I2	7.33	5.33	2.76E-07	4.33E-03
GRK1	7.33	5.33	2.76E-07	4.33E-03
SMIM10	6.88	4.94	2.02E-06	3.16E-02
CTSG	6.87	4.93	2.08E-06	3.26E-02

Table S7: Primer sequences

Target	Primer ID	Sequence (5' to 3')	Annealing T (°C)
MPO Exon 1	MPO Ex1 F	CTTCCTCTACCTCACCCCAC	62
	MPO Ex1 R	CTATCAGGCCCCAGAGCTAG	
MPO Exon 2	MPO Ex2 F	TTCCTAGCTCTGGGGCCT	62
	MPO Ex2 R	CCTCTCCCACCTTCAAGCT	
MPO Exon 3	MPO Ex3 F	CAAAGCCTTGCCTCTGTCTG	62
	MPO Ex3 R	TGGAGGAAGAAGTTGAGGGG	
MPO Exon 4-5	MPO Ex4-5 F	CCCCTCAACTTCTTCCTCCA	62
	MPO Ex4-5 R	TCAGCTGATCAGTGGGGAAG	
MPO Exon 6	MPO Ex6 F	GCCAGCTGATCTCCGTGT	62
	MPO Ex6 R	CAGCGTCTGGGAAAGGAAAC	
MPO Exon 7	MPO Ex7 F	CTGCTCATTAACCCTGCACC	62
	MPO Ex7 R	CCACAAGCTGCTCACAAACA	
MPO Exon 8	MPO Ex8 F	GGGGTTTCAGTGGAGCAAAT	62
	MPO Ex8 R	TCAACCCTCCCAACACCAAT	
MPO Exon 9	MPO Ex9 F	CCAAGAGCAGGCAGAGACT	64
	MPO Ex9 R	AGGCTAGAGAGTCAGACCAGA	
MPO Exon 10	MPO Ex10 F	TCTCGAATCCTCCTGACCCT	64
	MPO Ex10 R	TCTAATATGCTTTGGAGAGGGC	
MPO Exon 11	MPO Ex11 F	TCTCCAGTGACCTCCCCA	62
	MPO Ex11 R	AGGAGGAAATTTGGGCTCCA	
MPO Exon 12	MPO Ex12 F	ATATCCTGGGAGCAGCACAA	62
	MPO Ex12 R	CATTTTCTCAGCTGCACCCA	

Supplemental references

1. Navarini AA, Burden AD, Capon F, Mrowietz U, Puig L, Koks S, et al. European Consensus Statement on Phenotypes of Pustular Psoriasis. J Eur Acad Dermatol Venereol 2017:1792-9.
2. Mahil SK, Twelves S, Farkas K, Setta-Kaffetzi N, Burden AD, Gach JE, et al. AP1S3 Mutations Cause Skin Autoinflammation by Disrupting Keratinocyte Autophagy and Up-Regulating IL-36 Production. J Invest Dermatol 2016:2251-9.
3. Sidoroff A, Halevy S, Bavinck JN, Vaillant L, Roujeau JC. Acute generalized exanthematous pustulosis (AGEP)--a clinical reaction pattern. J Cutan Pathol 2001; 28:113-9.
4. Onoufriadis A, Simpson MA, Pink AE, Di Meglio P, Smith CH, Pullabhatla V, et al. Mutations in IL36RN/IL1F5 are associated with the severe episodic inflammatory skin disease known as generalized pustular psoriasis. Am J Hum Genet 2011; 89:432-7.
5. Setta-Kaffetzi N, Navarini AA, Patel VM, Pullabhatla V, Pink AE, Choon SE, et al. Rare Pathogenic Variants in IL36RN Underlie a Spectrum of Psoriasis-Associated Pustular Phenotypes. J Invest Dermatol 2013; 133:1366-9.
6. Li H, Handsaker B, Wysoker A, Fennell T, Ruan J, Homer N, et al. The Sequence Alignment/Map format and SAMtools. Bioinformatics 2009; 25:2078-9.
7. Wang K, Li M, Hakonarson H. ANNOVAR: functional annotation of genetic variants from high-throughput sequencing data. Nucleic Acids Res 2010; 38:e164.
8. Ruark E, Munz M, Renwick A, Clarke M, Ramsay E, Hanks S, et al. The ICR1000 UK exome series: a resource of gene variation in an outbred population. F1000Res 2015; 4:883.
9. Kircher M, Witten DM, Jain P, O'Roak BJ, Cooper GM, Shendure J. A general framework for estimating the relative pathogenicity of human genetic variants. Nat Genet 2014; 46:310-5.
10. Catapano M, Vergnano M, Romano M, Mahil SK, Choon SE, Burden AD, et al. Interleukin-36 promotes systemic Type-I IFN responses in severe psoriasis. Journal of Investigative Dermatology 2019; in press.
11. Canela-Xandri O, Rawlik K, Tenesa A. An atlas of genetic associations in UK Biobank. Nat Genet 2018; 50:1593-9.
12. Karczewski KJ, Francioli LC, Tiao G, Cummings BB, Alfoldi J, Wang Q, et al. Variation across 141,456 human exomes and genomes reveals the spectrum of loss-of-function intolerance across human protein-coding genes. BioRxiv 2019.
13. Stendahl O, Lindgren S. Function of granulocytes with deficient myeloperoxidase-mediated iodination in a patient with generalized pustular psoriasis. Scand J Haematol 1976; 16:144-53.

14. De Argila D, Dominguez JD, Lopez-Estebaranz JL, Iglesias L. Pustular psoriasis in a patient with myeloperoxidase deficiency. Dermatology 1996; 193:270.
15. Nguyen C, Katner HP. Myeloperoxidase deficiency manifesting as pustular candidal dermatitis. Clin Infect Dis 1997; 24:258-60.
16. Disdier P, Harle JR, Weiller-Merli C, Andrac L, Weiller PJ. Neutrophilic dermatosis despite myeloperoxidase deficiency. J Am Acad Dermatol 1991; 24:654-5.
