# Supplementary material for: Loss-of-Function Myeloperoxidase Mutations Are Associated with Increased Neutrophil Counts and Pustular Skin Disease
Source: Am J Hum Genet. 2020 Aug 5;107(3):539–43. doi: 10.1016/j.ajhg.2020.06.020 (PMC7477255; doi:10.1016/j.ajhg.2020.06.020)
Supplement: Document S2. Article plus Supplemental Information [file mmc2.pdf]

Loss-of-Function Myeloperoxidase Mutations Are Associated with Increased Neutrophil Counts and Pustular Skin Disease

Marta Vergnano,[1,2] Maja Mockenhaupt,[3,16] Natashia Benzian-Olsson,[1,16] Maren Paulmann,[3,16] Katarzyna Grys,[2] Satveer K. Mahil,[2] Charlotte Chaloner,[1] Ines A. Barbosa,[2] Suzannah August,[4] A. David Burden,[5] Siew-Eng Choon,[6] Hywel Cooper,[7] Alex A. Navarini,[8] Nick J. Reynolds,[9] Shyamal Wahie,[10] Richard B. Warren,[11] Andrew Wright,[12] The APRICOT and PLUM study team, Ulrike Huffmeier,[13] Patrick Baum,[14] Sudha Visvanathan,[15] Jonathan N. Barker,[2] Catherine H. Smith,[2] and Francesca Capon[1,17,*]

The identification of disease alleles underlying human autoinflammatory diseases can provide important insights into the mechanisms that maintain neutrophil homeostasis. Here, we focused our attention on generalized pustular psoriasis (GPP), a potentially life-threatening disorder presenting with cutaneous and systemic neutrophilia. Following the whole-exome sequencing of 19 unrelated affected individuals, we identified a subject harboring a homozygous splice-site mutation (c.2031−2A>C) in *MPO*. This encodes myeloperoxidase, an essential component of neutrophil azurophil granules. *MPO* screening in conditions phenotypically related to GPP uncovered further disease alleles in one subject with acral pustular psoriasis (c.2031−2A>C;c.2031−2A>C) and in two individuals with acute generalized exanthematous pustulosis (c.1705C>T;c.2031−2A>C and c.1552_1565del;c.1552_1565del). A subsequent analysis of UK Biobank data demonstrated that the c.2031−2A>C and c.1705C>T (p.Arg569Trp) disease alleles were also associated with increased neutrophil abundance in the general population ($p = 5.1 \times 10^{-6}$ and $p = 3.6 \times 10^{-5}$, respectively). The same applied to three further deleterious variants that had been genotyped in the cohort, with two alleles (c.995C>T [p.Ala332Val] and c.752T>C [p.Met251Thr]) yielding p values $< 10^{-10}$. Finally, treatment of healthy neutrophils with an MPO inhibitor (4-Aminobenzoic acid hydrazide) increased cell viability and delayed apoptosis, highlighting a mechanism whereby *MPO* mutations affect granulocyte numbers. These findings identify *MPO* as a genetic determinant of pustular skin disease and neutrophil abundance. Given the recent interest in the development of MPO antagonists for the treatment of neurodegenerative disease, our results also suggest that the pro-inflammatory effects of these agents should be closely monitored.

A tight regulation of neutrophil numbers is crucial to innate immune homeostasis. As mature granulocytes do not divide, their accumulation depends on the balance between progenitor proliferation, release of differentiated cells into the bloodstream, and clearance of aging cells.[1] Given the difficulty of manipulating primary neutrophils, the mechanisms that regulate these processes have mostly been investigated in animal models. In this context, the genetic characterization of human autoinflammatory diseases can provide crucial insights into the pathways that maintain neutrophil homeostasis.

Here we focused our attention on generalized pustular psoriasis (GPP [MIM: 614204]), a potentially life-threatening condition presenting with flares of neutrophilic skin inflammation (pustular eruptions), fever, increased production of acute phase reactants, and neutrophilia. While disease alleles have been described in *IL36RN*, *AP1S3*, and *CARD14*, the majority of affected individuals do not carry deleterious changes at these loci.[2]

To identify genetic determinants for GPP, we undertook whole-exome sequencing in 19 unrelated affected individuals of varying ancestry (Table S1, Figure 1A). Given the severity of the condition and the lack of parent-offspring transmissions, we hypothesized the presence of recessive loss-of-function alleles. We therefore filtered the variant profiles to retain rare homozygous changes predicted to cause premature protein truncation. This identified six candidate mutations, each affecting a single individual (Table S2).

The c.2031−2A>C substitution in *MPO* (MIM: 606989; GenBank: NM_000250) (Figure 1B) was selected for

[1]Department of Medical and Molecular Genetics, School of Basic and Medical Biosciences, King's College London, London SE1 9RT, UK; [2]St John's Institute of Dermatology, School of Basic and Medical Biosciences, King's College London, London SE1 9RT, UK; [3]Department of Dermatology, Medical Centre-University of Freiburg, Freiburg 79106, Germany; [4]Poole Hospital NHS Foundation Trust, Poole BH15 2JB, UK; [5]Department of Dermatology, University of Glasgow, Glasgow G12 8QQ, UK; [6]Department of Dermatology, Sultanah Aminah Hospital, Clinical School Johor Bahru, Monash University, Malaysia; [7]Portsmouth Dermatology Centre, St Marys Hospital, Portsmouth PO3 6AD, UK; [8]Department of Dermatology & Allergy, University Hospital of Basel, Basel 4031, Switzerland; [9]Translational and Clinical Research Institute, Newcastle University, Newcastle upon Tyne NE2 4HH, UK and Department of Dermatology and NIHR Newcastle Biomedical Research Centre, Newcastle Hospitals NHS Foundation Trust, Newcastle upon Tyne NE2 4LP, UK; [10]Department of Dermatology, University Hospital of North Durham, Durham DH1 5TW, UK; [11]Dermatology Centre, Salford Royal NHS Foundation Trust, Manchester NIHR Biomedical Research Centre, University of Manchester, Manchester M6 8HD, UK; [12]Centre for Skin Sciences, St Lukes Hospital, Bradford BD5 0NA, UK; [13]Institute of Human Genetics, Friedrich-Alexander-Universität Erlangen-Nürnberg, Erlangen 91054, Germany; [14]Boehringer-Ingelheim International GmbH, Biberach 88397, Germany; [15]Boehringer-Ingelheim Pharmaceuticals, Ridgefield, CT 06877, USA
[16]These authors contributed equally to this work
[17]Twitter: @FranciCapon
*Correspondence: francesca.capon@kcl.ac.uk
https://doi.org/10.1016/j.ajhg.2020.06.020.
© 2020 The Author(s). This is an open access article under the CC BY license (http://creativecommons.org/licenses/by/4.0/).

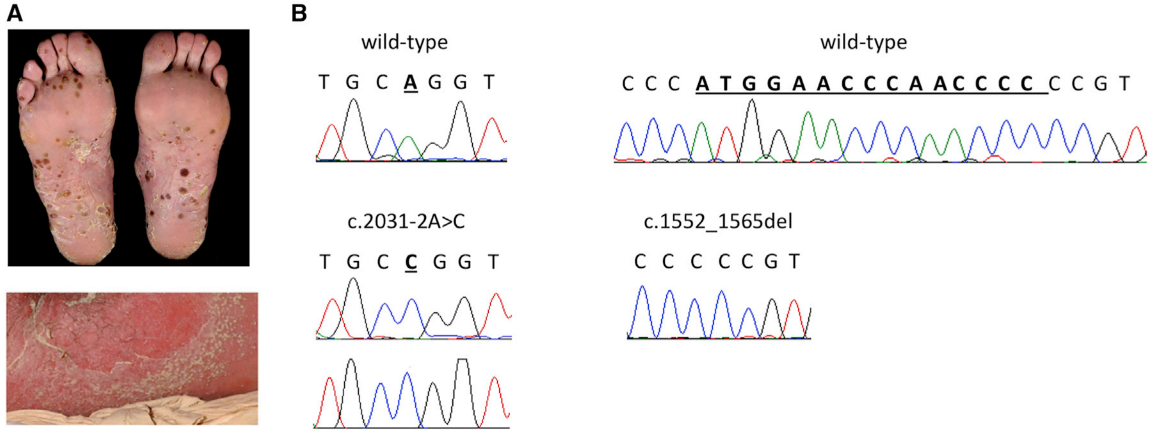

Figure 1. ***MPO*** **Mutations Are Associated with Pustular Skin Disease**
(A) Typical presentation of generalized pustular psoriasis (bottom panel, showing skin pustulation on an erythematous background) and acral pustular psoriasis (top panel, showing neutrophil-filled pustules affecting the soles).
(B) Validation of the disease alleles identified by whole-exome sequencing. The chromatograms show the c.2031−2A>C substitution observed in the GPP and APP subjects (left) and the c.1552_1565del deletion detected in a study participant with AGEP (right). The position of disease alleles is highlighted by bold, underlined font. Sanger sequencing could not be carried out in the c.2031−2A>C;c.1705C>T individual as no DNA was left for this subject.

follow-up, as the gene encodes myeloperoxidase, a major component of neutrophil azurophilic granules. Of note, the c.2031−2A>C change has been previously observed in individuals presenting with myeloperoxidase deficiency (MPOD [MIM: 254600]) (Table S3), an inherited defect of neutrophil microbicidal activity.[3] Specifically, Marchetti et al. demonstrated that the substitution affects splicing and leads to the production of a truncated protein lacking enzymatic activity.[3]

Here, the frequency of the c.2031−2C;c.2031−2C genotype among European GPP case subjects was much higher than that observed in the non-Finnish European exomes sequenced by the gnomAD consortium (5.3% versus 0.003%; $p = 0.001$) (Table 1). Given that frequency estimates obtained in small datasets are not always robust, the *MPO* coding region was next examined in a validation sample, including 14 GPP subjects and 109 individuals with acral variants of pustular psoriasis (APP). This uncovered a further study participant harboring a homozygous c.2031−2A>C substitution (Figure 1B, Table 2, and Supplemental Note).

While no other bi-allelic or truncating *MPO* changes were detected (Table S4), a comparison of the replication cohort against a second gnomAD dataset (32,264 Non-Finnish European genomes) confirmed the elevated frequency of the c.2031−2C;c.2031−2C genotype in case subjects versus control subjects (0.8% versus 0%; $p = 0.004$) (Table 1). Finally, the analysis of the combined study resource (142 case subjects versus 89,010 control subjects) yielded a p value of 1.5×10^{-5} (Table 1). Importantly, the c.2031−2C;c.2031−2C genotype was also absent from 590 British exomes processed with our in-house pipeline. Thus, the association with GPP/-APP is unlikely to be a technical artifact or to reflect population stratification between case and control subjects.

To further investigate the impact of *MPO* recessive alleles, we queried exome-sequencing data available for 96 unrelated individuals affected by acute generalized exanthematous pustulosis (AGEP). This is a severe cutaneous adverse reaction, which can be triggered by drugs (mostly antibiotics and antifungals), leading to flares of skin pustulation, fever, and systemic neutrophilia.[4] Our analysis identified an affected individual, who was homozygous for a 14 bp *MPO* deletion (c.1552_1565del [p.Met519Profs*21]). A second study participant had inherited the c.2031−2A>C substitution previously observed in GPP/APP, in conjunction with a damaging c.1705C>T (p.Arg569Trp) change (CADD score: 35.0) (Table 2, Supplemental Note).

Of note, homozygous c.1705C>T (p.Arg569Trp) mutations have been repeatedly observed in individuals affected by MPOD.[5,6] A c.1555_1568del variant, which overlaps the c.1552_1565del variant described here, has also been documented in an affected-relative pair, where it triggered nonsense-mediated decay in at least one individual[7] (Table S3). Thus, all the *MPO* alleles observed in our dataset have a well-established impact on protein function.

MPOD is a mild immune deficiency that is clinically well characterized. We therefore undertook a systematic literature review, to better understand the connection between *MPO* mutations, MPOD, and skin pustulation. We examined 28 articles describing the presentation of MPOD in 217 individuals. This uncovered four case reports where the disease manifested with pustular eruptions and a fifth where it was associated with the severe neutrophilic dermatosis known as pyoderma gangrenosum (Table S5). Given the very low prevalence of the above conditions (<1:100,000), these observations strengthen the link between MPO dysfunction and neutrophilic inflammation.

Table 1. Frequency of the c.2031−2C;c.2031−2C Genotypes in Case Subjects versus Control Subjects

	Genotype Counts (%)		
	**Cases**	**Controls**	**p Value**
Discovery cohort	1/19 (5.3%)	2/56,7466 (0.003%)	0.001
Replication cohort	1/123 (0.8%)	0/32,264 (0%)	0.004
Combined study cohort	2/142 (1.4%)	2/89,010 (0.002%)	1.5×10^{-5}

c.2031−2A>C was the only truncating change observed in the homozygous state in the control subjects, obviating the need for a burden association test.

To investigate the mechanisms whereby *MPO* mutations contribute to disease, we explored the phenotypic effects of the c.2031−2A>C variant though a Phenome-Wide Association study (PheWAS). We queried the UK Biobank dataset, which includes genotype information and health data for a well-characterized population cohort (>450,000 individuals).[8] While the fraction of c.2031−2C homozygotes (0.003%) (Table S3) present in the biobank was very small, c.2031−2A>C heterozygotes accounted for approximately 1% of study participants. Thus, we were able to analyze the c.2031−2A>C genotypes against 778 available phenotypes. This revealed that the traits showing the most significant associations with c.2031−2A>C were related to leukocyte counts. In this context, the largest effect size (beta) was observed for the association with neutrophil abundance (beta = 0.45; $p = 5.1 \times 10^{-6}$; Figure 2A). To validate these findings, we examined the c.1705C>T (p.Arg569Trp) change and three additional MPOD alleles (c.518A>G [p.Tyr173Cys], c.752T>C [p.Met251Thr], c.995C>T [p.Ala332Val]) for which genotype data were available in UK Biobank (Table S3). We found that all were associated with increased neutrophil accumulation, with p values ranging from 0.008 to 3.9×10^{-28} (Figure 2B).

To explore the pathways underlying the effects of *MPO* alleles on granulocyte numbers, we examined RNA-sequencing profiles generated in pure neutrophil populations (see Supplemental Subjects and Methods). Specifically, we compared gene expression in the c.2031−2A>C homozygous GPP individual versus 11 healthy control subjects. We observed that *MPO* was expressed at comparable levels in the affected subject and the unaffected control subjects (FDR > 0.5). As c.2031−2A>C affects the splicing of the last exon, this is in keeping with the expectation of an escape from nonsense-mediated decay.

Conversely, we found that 95 genes were upregulated in the affected individual (FDR < 0.05) (Table S6). The majority of these loci (85/95) were not overexpressed in 7 unrelated individuals with GPP (all *MPO* wild type) examined in parallel, indicating that the changes are unlikely to be a secondary effect of inflammation.

While the experiment was limited by the modest sample size and the number of differentially expressed genes was too small for pathway enrichment analyses, we noted that two of the five most upregulated loci (*PBK* and *GUCYA2*) encode proteins (PDZ binding kinase and soluble guanylate cyclase alpha-2 subunit) that can inhibit apoptosis.[9,10] This suggests that *MPO* mutations may affect neutrophil survival.

We investigated this possibility by using a myeloperoxidase inhibitor (4-Aminobenzoic acid hydrazide, ABAH) to mimic the effects of *MPO* disease alleles in cell culture experiments. We induced neutrophil apoptosis through Phorbol 12-myristate 13-acetate (PMA) stimulation and assessed the effects of ABAH pre-treatment on this process. While PMA caused substantial neutrophil death, we found that ABAH supplementation caused an increase in cell viability (Figure 2C) and a reduction in the number of apoptotic cells (Figure 2D). Thus, neutrophil apoptosis is downregulated in the absence of MPO activity.

Our findings (and those independently reported by Haskamp et al.[11]) demonstrate a significant association between *MPO* mutations and pustular skin disease. While the disease alleles described here have also been implicated in MPOD, we did not observe any evidence of immune deficiency in the individuals examined in this study. Likewise, pustular skin disease is present in only a fraction of people affected by MPOD. Thus, it is tempting to speculate that the manifestations of *MPO* mutations

Table 2. Disease Features Observed in Individuals with Bi-allelic *MPO* Mutations

Subjects	Sex	Age of Onset	Diagnosis[a]	Systemic Involvement	Genotype
GYFAP0014	F	36	GPP	fever and neutrophilia	c.2031−2A>C;c.2031−2A>C
DDPLM0001	F	24	APP	–	c.2031−2A>C;c.2031−2A>C
SCAR2124	F	80	AGEP (methotrexate)	fever and neutrophilia	c.1552_1565del;c.1552_1565del
SCAR2567	F	69	AGEP (hydroxychloroquine)	fever	c.2031−2A>C;c.1705C>T

None of the affected individuals reported a history of recurrent infections.
[a]The most likely culprit drug for each AGEP subject is reported in parentheses. AGEP, acute generalized exanthematous pustulosis; APP, acral pustular psoriasis; GPP, generalized pustular psoriasis.

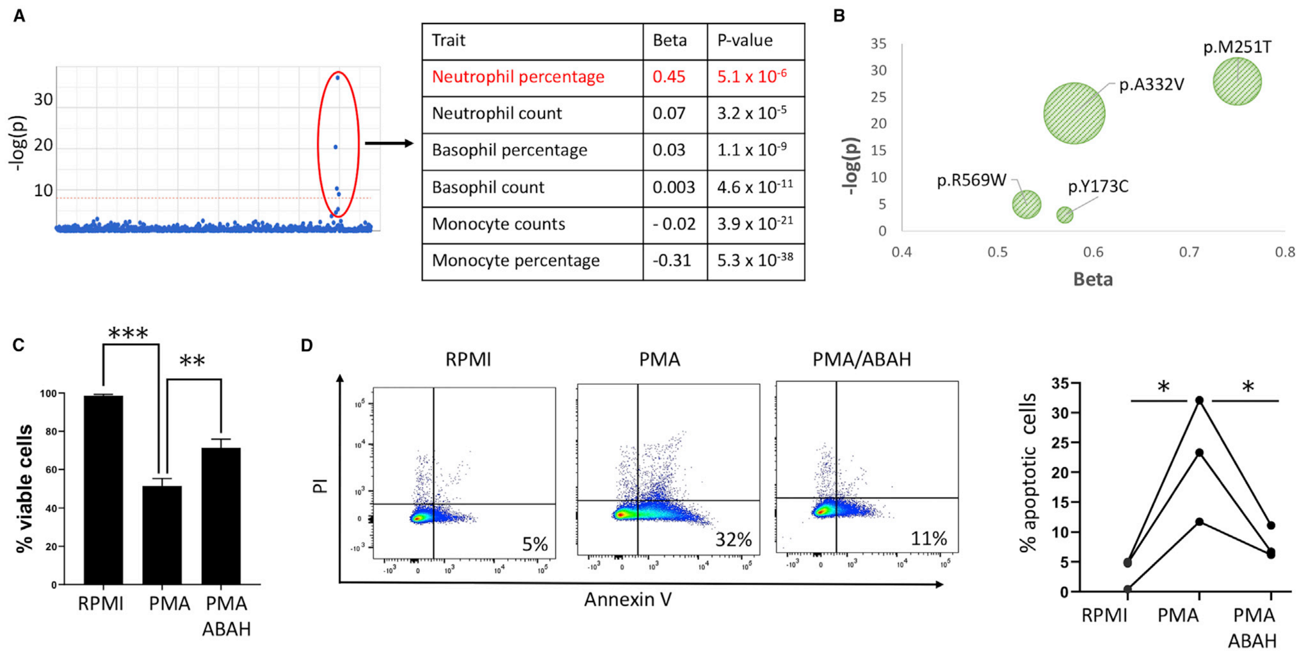

Trait	Beta	P-value
Neutrophil percentage	0.45	5.1×10^{-6}
Neutrophil count	0.07	3.2×10^{-5}
Basophil percentage	0.03	1.1×10^{-9}
Basophil count	0.003	4.6×10^{-11}
Monocyte counts	- 0.02	3.9×10^{-21}
Monocyte percentage	-0.31	5.3×10^{-38}

Figure 2. *MPO* Mutations Are Associated with Increased Neutrophil Counts and Delayed Apoptosis

(A) Manhattan plot where each dot represents the association between c.2031−2A and a clinical trait. The p values for phenotypes related to leukocyte counts are highlighted with a red circle and reported on the right, alongside the effect sizes (beta).

(B) Association between MPO deficiency alleles and neutrophil percentage. The size of each bubble represents the frequency of the mutation in UK Biobank.

(C and D) ABAH pre-treatment of cells stimulated with PMA increases viability (C) and downregulates apoptosis (D). In (C) data are presented as the mean (±SD) of four experiments carried out in triplicate. In (D) each line represents an independent healthy donor. A representative set of flow cytometry plots is shown on the left with the percentage of apoptotic cells (AnnexinV$^+$, PI$^-$ population) for each condition. RPMI = untreated (medium only); *$p < 0.05$ **$p < 0.01$; ***$p < 0.001$ (non-parametric ANOVA).

may be influenced by background polygenic variation, especially as a similar phenomenon has been documented in rare hematological phenotypes.[12] Of note, the c.2031−2C;c.2031−2C GPP individual described here also harbored a deleterious *AP1S3* allele (GenBank: NM_001039569.2; c.97C>T [p.Arg33Trp]),[13] which further supports the involvement of genetic modifiers.

An increased prevalence of spondyloarthropathy has also been reported among individuals with MPO deficiency.[14] This suggests that the disruption of neutrophil apoptosis may affect immune homeostasis in multiple organs. Given that MPO inhibitors are being developed for the treatment of neurodegenerative disease,[15] our data suggest that the inflammatory side effects of these agents should be closely monitored during clinical trials.

The results of our PheWAS indicate that the effects of *MPO* alleles are likely to be mediated by a systemic upregulation of neutrophil numbers. Of note, a significant association between granulocyte abundance and a common *MPO* variant has previously been documented,[16] further supporting the role of the gene in neutrophil homeostasis.

Further studies will be required to dissect the molecular mechanisms whereby MPO deficiency downregulates cell death. Given that *PBK* (one of the most upregulated genes in the c.2031−2A>C homozygous individual) is an inhibitor of myeloid cell apoptosis,[9] its role is worthy of further examination. A proposed link between MPO-related oxidative stress, NF-κB activation, and apoptotic signaling[17] should also be investigated. While experimentally demanding, these studies have the potential to illuminate key regulators of innate immune homeostasis and uncover new candidate genes for neutrophilic conditions.

Data and Code Availability

The neutrophil RNA-sequencing dataset described in this study may be obtained from the Gene Expression Omnibus using identifier GSE123787.

Supplemental Data

Supplemental Data can be found online at https://doi.org/10.1016/j.ajhg.2020.06.020.

Acknowledgments

This research has been conducted using the UK Biobank Resource. We are grateful to Athanasios Niaouris and Nick Dand for their technical and analytical support. We acknowledge support from the Department of Health via a National Institute of Health Research (NIHR) Biomedical Research Centre award to Guy's and St Thomas' NHS Foundation Trust in partnership with King's College London and King's College Hospital NHS Foundation Trust (guysbrc-2012-1). We also acknowledge support from the

Newcastle NIHR Biomedical Research Centre. The APRICOT trial is funded by the Efficacy and Mechanism Evaluation Programme (grant EME 13/50/17). This work has been partly funded by a European Academy of Dermatology and Venereology (EADV) award to J.N.B. and F.C. (grant PPRC-2018-25). U.H. is funded by the DFG (CRC1181, Project A05). M.V. is supported by a Medical Research Council (MRC) PhD studentship, N.B.-O. by a NIHR pre-doctoral fellowship (NIHR300473), and S.K.M. by an MRC Clinical Academic Research Partnership award (MR/T02383X/1). R.B.W. is supported by the Manchester NIHR Biomedical Research Centre. N.J.R. is a NIHR senior investigator. He also acknowledges support from the Newcastle MRC/EPSRC Molecular Pathology Node and the Newcastle NIHR Medtech and In vitro diagnostic Co-operative. M.M. and M.P. are part of The International Registry of Severe Cutaneous Adverse Reaction (RegiSCAR) Consortium, funded by unrestricted grants from the European Commission (QLRT-2002-01738), GIS-Institut des Maladies Rares and INSERM (4CH09G) in France, and by a consortium of pharmaceutical companies.

Declaration of Interests

A.D.B. has received funding from Boehringer-Ingelheim. C.H.S. has been a principal (or co-) investigator on commercially sponsored clinical trials and investigator-led studies funded by AbbVie, GaxoSmithKline, Janssen, Novartis, Pfizer, Regeneron, Roche, Sanquin, Celgene, Sanofi, LEO Pharma, Boehringer Ingleheim, and UCB Pharma. F.C. has received funding from Boehringer-Ingelheim and consultancy fees from AnaptysBio. J.N.B. has received funding and fees from Abbvie, Boehringer-Ingelheim, Bristol Myers Squibb, Celegene, Ely Lily, Novartis, Pfizer, Samsung, Sienna, and Sun Pharma. N.J.R. has received research funding for clinical trials from AnaptysBio through Newcastle Hospitals NHS Foundation Trust. P.B. and S.V. are Boehringer-Ingelheim employees. S.W. has received non-financial support (sponsorship to attend dermatology conferences) from Janssen, Abbvie, Novartis, and Almirall.

Received: February 26, 2020
Accepted: June 23, 2020
Published: August 5, 2020

Web Resources

CADD, https://cadd.gs.washington.edu/
ClinVar, https://www.ncbi.nlm.nih.gov/clinvar/
EGA, https://www.ebi.ac.uk/ega/home
GeneAtlas, http://geneatlas.roslin.ed.ac.uk/
Gene Expression Omnibus, https://www.ncbi.nlm.nih.gov/gds
gnomAD server, https://gnomad.broadinstitute.org/
OMIM, https://omim.org/
PubMed, https://www.ncbi.nlm.nih.gov/pubmed/

References

1. Nicolás-Ávila, J.A., Adrover, J.M., and Hidalgo, A. (2017). Neutrophils in Homeostasis, Immunity, and Cancer. Immunity *46*, 15–28.
2. Twelves, S., Mostafa, A., Dand, N., Burri, E., Farkas, K., Wilson, R., Cooper, H.L., Irvine, A.D., Oon, H.H., Kingo, K., et al. (2019). Clinical and genetic differences between pustular psoriasis subtypes. J. Allergy Clin. Immunol. *143*, 1021–1026.
3. Marchetti, C., Patriarca, P., Solero, G.P., Baralle, F.E., and Romano, M. (2004). Genetic characterization of myeloperoxidase deficiency in Italy. Hum. Mutat. *23*, 496–505.
4. Sidoroff, A., Halevy, S., Bavinck, J.N., Vaillant, L., and Roujeau, J.C. (2001). Acute generalized exanthematous pustulosis (AGEP)–a clinical reaction pattern. J. Cutan. Pathol. *28*, 113–119.
5. Kizaki, M., Miller, C.W., Selsted, M.E., and Koeffler, H.P. (1994). Myeloperoxidase (MPO) gene mutation in hereditary MPO deficiency. Blood *83*, 1935–1940.
6. Nauseef, W.M., Brigham, S., and Cogley, M. (1994). Hereditary myeloperoxidase deficiency due to a missense mutation of arginine 569 to tryptophan. J. Biol. Chem. *269*, 1212–1216.
7. Romano, M., Dri, P., Da Dalt, L., Patriarca, P., and Baralle, F.E. (1997). Biochemical and molecular characterization of hereditary myeloperoxidase deficiency. Blood *90*, 4126–4134.
8. Sudlow, C., Gallacher, J., Allen, N., Beral, V., Burton, P., Danesh, J., Downey, P., Elliott, P., Green, J., Landray, M., et al. (2015). UK biobank: an open access resource for identifying the causes of a wide range of complex diseases of middle and old age. PLoS Med. *12*, e1001779.
9. Liu, Y., Liu, H., Cao, H., Song, B., Zhang, W., and Zhang, W. (2015). PBK/TOPK mediates promyelocyte proliferation via Nrf2-regulated cell cycle progression and apoptosis. Oncol. Rep. *34*, 3288–3296.
10. Weissmann, N., Lobo, B., Pichl, A., Parajuli, N., Seimetz, M., Puig-Pey, R., Ferrer, E., Peinado, V.I., Domínguez-Fandos, D., Fysikopoulos, A., et al. (2014). Stimulation of soluble guanylate cyclase prevents cigarette smoke-induced pulmonary hypertension and emphysema. Am. J. Respir. Crit. Care Med. *189*, 1359–1373.
11. Haskamp, S., Bruns, H., Hahn, M., Hoffmann, M., Gregor, A., Löhr, S., Hahn, J., Schauer, C., Ringer, M., Flamann, C., et al. (2020). Myeloperoxidase modulates inflammation in generalized pustular psoriasis and additional rare pustular skin diseases. Am. J. Hum. Genet. *107*. Published online August 5, 2020. https://doi.org/10.1016/j.ajhg.2020.06.020.
12. Vuckovic, D., Bao, E.L., Akbari, P., Lareau, C., Moussas, A., Jiang, T., Chen, M.H., Raffield, L.M., Tardaguila, M., Huffman, J.E., et al. (2020). The Polygenic and Monogenic Basis of Blood Traits and Diseases. medRxiv. https://doi.org/10.1101/2020.02.02.20020065.
13. Setta-Kaffetzi, N., Simpson, M.A., Navarini, A.A., Patel, V.M., Lu, H.C., Allen, M.H., Duckworth, M., Bachelez, H., Burden, A.D., Choon, S.E., et al. (2014). AP1S3 mutations are associated with pustular psoriasis and impaired Toll-like receptor 3 trafficking. Am. J. Hum. Genet. *94*, 790–797.
14. Kutter, D., Devaquet, P., Vanderstocken, G., Paulus, J.M., Marchal, V., and Gothot, A. (2000). Consequences of total and subtotal myeloperoxidase deficiency: risk or benefit? Acta Haematol. *104*, 10–15.
15. Galijasevic, S. (2019). The development of myeloperoxidase inhibitors. Bioorg. Med. Chem. Lett. *29*, 1–7.
16. Astle, W.J., Elding, H., Jiang, T., Allen, D., Ruklisa, D., Mann, A.L., Mead, D., Bouman, H., Riveros-Mckay, F., Kostadima, M.A., et al. (2016). The Allelic Landscape of Human Blood Cell Trait Variation and Links to Common Complex Disease. Cell *167*, 1415–1429.e19.
17. Kanayama, A., and Miyamoto, Y. (2007). Apoptosis triggered by phagocytosis-related oxidative stress through FLIPS down-regulation and JNK activation. J. Leukoc. Biol. *82*, 1344–1352.

The American Journal of Human Genetics, Volume *107*

Supplemental Data

Loss-of-Function Myeloperoxidase Mutations Are Associated with Increased Neutrophil Counts and Pustular Skin Disease

Marta Vergnano, Maja Mockenhaupt, Natashia Benzian-Olsson, Maren Paulmann, Katarzyna Grys, Satveer K. Mahil, Charlotte Chaloner, Ines A. Barbosa, Suzannah August, A. David Burden, Siew-Eng Choon, Hywel Cooper, Alex A. Navarini, Nick J. Reynolds, Shyamal Wahie, Richard B. Warren, Andrew Wright, The APRICOT and PLUM study team, Ulrike Huffmeier, Patrick Baum, Sudha Visvanathan, Jonathan N. Barker, Catherine H. Smith, and Francesca Capon

Supplemental note: Membership of the PLUM and APRICOT study team

The following members of the PLUM and APRICOT study team contributed to this work:

Thamir Abraham (Peterborough city Hospital),Mahmud Ali (Worthing Hospital), Suzannah August (Poole Hospital), David Baudry (Guy's Hospital, London), Anthony Bewley (Whipps Cross University Hospital, London), Hywel Cooper (St Marys Hospital, Portsmouth), John Ingram (University Hospital of Wales), Susan Kelly (The Royal Shrewsbury Hospital), Mohsen Korshid (Basildon Hospital), Effie Ladoyanni (Russell's Hall Hospital, Dudley), John McKenna (Leicester Royal Infirmary),Freya Meynell (Guy's Hospital, London), Richard Parslew (Broadgreen Hospital, Liverpool), Prakash Patel (Guy's Hospital, London), Angela Pushparajah (Guy's Hospital, London), Nick Reynolds (Newcastle Hospitals), Catherine Smith (Guy's Hospital, London), Shyamal Wahie (University Hospital of North Durham and Darlington Memorial Hospital), Richard Warren (Salford Royal Infirmary), Andrew Wright (St Lukes Hospital, Bradford).

Additional affected individuals were recruited by A David Burden (Glasgow Western Infirmary), Siew-Eng Choon (Johor Bahru Hospital, Malaysia), Brian Kirby (St Vincent University Hospital, Dublin, Ireland), Alexander Navarini (University Hospital Zurich, Switzerland) and Marieke Sieger (Radboud Medical Centre, Nijmegen, The Netherlands).

Supplemental Note: Case Reports

The four individuals harbouring bi-allelic *MPO* mutations are described below.

GYFAP0014 is a British 54-year-old female of European descent. She was diagnosed with Acrodermatitis Continua of Hallopeau (ACH, a localised pustular eruption affecting the nails and distal phalanxes) aged 10. She also has a history of generalised pustular psoriasis that flared during pregnancy (at age 36 and 39) with widespread pustulation, high-fever, severe neutrophilia (>15,000 cells/mm^3) and elevated CRP levels (>100mg/L). Following several years of quiescent disease on infliximab infusions (5mg per kg every 10 weeks) and oral methotrexate (5mg per week) co-therapy, both treatments were withdrawn at age 52. Skin clearance has subsequently been maintained (2 years follow up to date). Her comorbidities include anxiety and depression. She has a body mass index (BMI) of 30. She does not have concurrent plaque psoriasis or psoriatic arthritis, and there is no family history of psoriasis.

DDPLM001 is a 35-year-old British female of European descent. She has been suffering from palmoplantar pustulosis since she was 24. She also has ACH with nail loss on her left hand and is affected by chronic plaque psoriasis and psoriatic arthritis. Her BMI is 26 and she is an ex-smoker. She has been receiving ciclosporin for the past 3 years with good effect. Her previous treatments include methotrexate and oral PUVA. There is no family history of psoriasis.

SCAR2124 is a female from Germany who presented with acute generalised exanthematous pustulosis (AGEP) at age 80, requiring an admission to hospital for 23 days. She suffered from widespread pustulation, fever and severe neutrophilia (>15,000 cells/mm^3). The culprit drugs (in order of suspicion) are methotrexate, pantoprazole and sultamicillin (ampicillin/sulbactam).

SCAR2567 is a female from Germany who presented with AGEP secondary to hydroxychloroquine at age 70. She suffered from high fever and pustulation, requiring an admission to hospital for 5 days. Her comorbidities include previous giant cell arteritis.

Supplemental Subjects and Methods

Study participants

The study was carried out according to the principles of the declaration of Helsinki and was approved by the ethics committees of participating institutions. Written informed consent was also obtained from all participants. Ninety-four individuals with generalised or acral forms of pustular psoriasis were ascertained through the APRICOT clinical trial (Anakinra in Pustular psoriasis, Response In a Controlled Trial; EudraCT n. 2015-003600-23) and its sister mechanistic study PLUM (Pustular psoriasis, eLucidating Underlying Mechanisms). Forty-eight additional cases were recruited outside of these programmes at St John's Institute of Dermatology (London, UK), Glasgow Western Infirmary (UK), Zurich University Hospital (Switzerland) and Hospital Sultanah Aminah (Johor Bahru, Malaysia). Pustular psoriasis was diagnosed by expert dermatologists, based on clinical examination and/or published consensus criteria[1]. Given various reports of digenic inheritance in GPP[2], individuals harbouring deleterious alleles at known disease loci were not excluded from the study cohort.

Subjects affected by AGEP were actively recruited by a network of hospitals in six countries. All were interviewed by trained investigators who recorded detailed information on the clinical course of the disease, previous medical history and suspected causative factors including infections and medications. Drug intake in the month before hospital admission was also recorded in a systematic way. All case notes were reviewed by a multinational expert committee of dermatologists blinded for information on risk factors. Clinical photographs were viewed together with clinical information from the case record forms and histological information. Cases were then assessed with a published scoring system[3] and were either excluded from the cohort or classified as definite, probable or possible. Only definite and probable cases were included in this study.

Given previous evidence of digenic inheritance in pustular skin disorders, affected individuals were included in the study, even if they harboured mutations at known disease loci[2, 4].

Healthy volunteers for MPO inhibition studies were recruited among the personnel of St John's Institute of Dermatology.

Whole-exome sequencing and Sanger sequencing

Five affected individuals were exome sequenced as part of previously published studies[4, 5]. For the remaining samples libraries were prepared with the Agilent SureSelect Human All Exome kit and run on an Illumina HiSeq3000 instrument. Paired-end reads were aligned to the hg19 reference genome with Novoalign (Novocraft Technologies) After the removal of duplicate reads, SNPs and small insertion deletions were identified with SAMtools[6]. Finally, variant files were annotated with ANNOVAR[7]. The same pipeline was used to process 590 control exomes generated by the UK Institute of Cancer Research[8].

Given that none of the affected individuals had an affected parent, exome profiles were filtered based on an autosomal recessive mode of inheritance. To maximise the likelihood of detecting deleterious loss-of-function alleles, variants were only retained if they: i) caused splicing, stop-gain, stop-loss and frameshift changes; ii) occurred in homozygosis; iii) had a MAF ≤ 0.01 in the ExAC dataset and in our in-house sequencing database; iv) were associated with CADD pathogenicity scores>15[9].

Upon completion of the filtering process, the *MPO* coding region was screened by Sanger sequencing in 14 additional GPP subjects, using the primers listed in Table S7. The gene was also examined in 109 APP sufferers, by interrogating whole-exome data generated as described above.

Systematic literature review

The PubMed database was queried using the terms (MPO deficiency) or (myeloperoxidase deficiency). The search, which was restricted to articles written in English and published before June 2019, retrieved 225 papers. Following the removal of reviews and irrelevant studies (e.g. characterisation of animal models and cell lines), 28 original articles were examined in detail.

Neutrophil RNA-sequencing

Neutrophil RNA-sequencing of 8 unrelated GPP individuals and 11 healthy controls was carried out as part of a previous study[10]. Briefly, the MACSxpress Whole Blood Neutrophil Isolation Kit (Miltenyi Biotec) was used to isolate high-purity (>95%) neutrophil populations. Following mRNA capture, samples were sequenced on a NextSeq 500 Illumina platform. Reads were then aligned to the HG38 genome and quantified with HTseq-count[10].

Here, the expression profile of the individual carrying the c.2031-2A>C variant was compared to that of the 11 controls. Fold changes (FC) were calculated by dividing the gene expression levels (RPKM) observed in the proband by the mean RPKM of the controls. FC values were then converted into z-scores by normalising their distribution to a mean of zero and standard deviation of 1. Finally, p-values were computed based on the normal distribution and adjusted for multiple testing (Bonferroni correction). The specificity of the gene expression changes was assessed through a parallel analysis of the 7 remaining GPP subjects, which were compared to the 11 healthy controls.

Cell viability and apoptosis assays

Neutrophils were purified using the MACSxpress Whole Blood Neutrophil Isolation Kit (Miltenyi) and 10^6 cells were cultured in 500µl RPMI (Gibco) supplemented with 1% BSA (Sigma-Aldrich). For viability assays, cultures were treated with 400µM ABAH (Abcam) for 4h after which 100nM PMA (Cayman Chemicals) or vehicle was added to the medium. After 20h, live cells were counted with a Nucleocounter NC-200 (ChemMetec). For apoptosis assays, cultures were treated with 400µM ABAH for 4h after which 25nM PMA or vehicle was added to the medium. After 20h, cells were processed

with the Annexin-V-FLUOS Staining Kit (Sigma Aldrich) and analysed on a BD FACSCanto™ II Flow Cytometry System. All experiments were carried out three times.

Analysis of UK Biobank dataset

The phenotypes associated with the c.2031-2A>C mutation were identified with the PheWAS function of The Gene ATLAS browser (a database reporting the results of 778 genome-wide association studies, carried out in UK Biobank using an additive genetic model[11]). To validate the association between c.2031-2A>C and neutrophil count variation, the details of additional MPOD alleles were retrieved from the OMIM database and used in further Gene ATLAS queries.

Statistical tests

The frequency of the c.2031-2A>C/c.2031-2A>C genotype was compared in cases vs. gnomAD controls[12] (non-Finnish European exomes v.2.1.1 and non-Finnish European genomes v.3), using Fisher's exact test. The effects of ABAH on PMA treated cells were assessed with a non-parametric ANOVA (Friedman's test).

Supplemental Tables

Table S1: Composition of the study cohort

Diagnosis	Sex	Ancestry	Average age of onset
GPP-WES (n=19)	4 males<br>15 females	European (n=9); Asian (n=9); Romani (n=1)	31 years
GPP-Sanger (n=14)	3 males<br>11 females	European (n=10); Asian (n=4)	40 years
APP (n=109)	22 males<br>87 females	European (n=109)	44 years

APP, Acral Pustular psoriasis; GPP, Generalized Pustular Psoriasis; WES, whole-exome sequencing

Table S2: Rare loss-of-function variants identified in 19 unrelated GPP individuals

Gene	Variant	Global MAF	Pathogenicity score (CADD)[1]	Gene function
ARAP1	c.4070+5G>T	0.0005	22.8	Modulates actin cytoskeleton remodelling
CCSER2	c.1868+5T>A	0.0003	15.8	Might play a role in microtubule bundling
DMBT1	c.1504C>T	0.00002	35.0	Candidate tumour suppressor gene for brain, lung, oesophageal, gastric, and colorectal cancers
EIF4G1	c.698-3C>T	0.0016	16.5	Encodes a component of the EIF4F protein complex
FAM83A	c.871C>T	0.0018	36.0	Probable proto-oncogene activating AKT/TOR signalling
MPO	**c.2031-2A>C**	**0.0043**	**32.0**	**Major component of azurophilic granules; produces hypohalous acids that are essential to the microbicidal activity of neutrophils**

[1]CADD scores>15.0 are considered as evidence of pathogenicity; MAF, minor allele frequency

Table S3: Myeloperoxidase deficiency alleles reported in the OMIM database

cDNA (protein) change	Clinvar accession n.	dbSNP ID	UK Biobank MAF (n. of homozygotes; %)
c.2031-2A>C (p.Phe678_Ser745delins(71))	VCV000003632	rs35897051	0.0064 (14; 0.003%)
c.1715T>G (p.Leu572Trp)	VCV000003631	rs119469012	n/a
c.1705C>T (p.Arg569Trp)	VCV000003626	rs119468010	0.0035 (4; 0.001%)
c.1555_1568del (p.Met519fs)	VCV000003629	rs536522394	n/a
c.1501G>A (p.Gly501Ser)	VCV000003634	rs119469013	n/a
c.1495C>T (p.Arg499Cys)	VCV000003635	rs119469014	n/a
c.995C>T (p.Ala332Val)	VCV000003630	rs28730837	0.016 (121; 0.03%)
c.752T>C (p.Met251Thr)	VCV000003628	rs56378716	0.013 (76; 0.02%)
c.518A>G (p.Tyr173Cys)	VCV000003627	rs78950939	0.0012 (1; 0.0002%)

MAF, minor allele frequency; n/a: not available

Table S4: Rare and low-frequency *MPO* variants observed in the replication cohort[1]

cDNA change	Protein change	dbSNP ID	MAF among gnomAD controls	MAF among cases	Mutation class (status)
c.752T>C	p.Met251Thr	rs56378716	0.014	0.012	missense (heterozygous)
c.995C>T	p.Ala332Val	rs28730837	0.018	0.016	missense (heterozygous)
c.1379G>A	p.Arg460Gln	rs149133270	0.002	0.004	missense (heterozygous)
c.1643G>A	p.Arg548Gln	rs144371238	0.0001	0.004	missense (heterozygous)
c.1705C>T	p.Arg569Trp	rs119468010	0.003	0.004	missense (heterozygous)
c.2149A>G	p.Ile717Val	rs2759	0.029	0.032	missense (heterozygous)

[1]Excluding the c.2031-2A>C allele, which is described in the main text

Table S5: Association of pustular skin disease and myeloperoxidase deficiency

Case report	*Reference*
• 46-year-old male with MPOD, plaque psoriasis and GPP.<br>• Identical twin with mild pustular psoriasis.	Stendahl and Lindgren[13]
• 61-year-old female with MPOD and annular pustular psoriasis	De Argila et al.[14]
• 20-year-old male with MPOD developed generalized pustular eruptions following injury	Nguyen and Katner[15]
• 81-year-old female with MPOD and pyoderma gangrenosum	Disdier et al.[16]

GPP, Generalized pustular psoriasis; MPOD, myeloperoxidase deficiency

Table S6: Genes upregulated in the neutrophils of the c.2031-2A>C homozygous GPP individual

Gene	fold change	z-score	p value	adj p value
PBK	33.00	27.02	1.06E-159	1.66E-155
GPR33	33.00	27.02	1.06E-159	1.66E-155
COL14A1	27.50	22.37	7.88E-110	1.24E-105
GUCY1A2	22.00	17.72	2.40E-69	3.77E-65
SLC10A4	22.00	17.72	2.40E-69	3.77E-65
DEFB1	22.00	17.72	2.40E-69	3.77E-65
DPP10	22.00	17.72	2.40E-69	3.77E-65
PURG	22.00	17.72	2.40E-69	3.77E-65
CALCB	22.00	17.72	2.40E-69	3.77E-65
DUOXA1	22.00	17.72	2.40E-69	3.77E-65
BDKRB2	22.00	17.72	2.40E-69	3.77E-65
DEFA3	21.56	17.35	1.65E-66	2.58E-62
METTL7B	15.40	12.15	3.71E-33	5.82E-29
NNMT	15.40	12.15	3.71E-33	5.82E-29
SLC22A16	13.17	10.26	5.67E-24	8.89E-20
CES1	13.02	10.13	2.07E-23	3.24E-19
USP2	11.00	8.43	1.53E-16	2.40E-12
C11orf45	11.00	8.43	1.53E-16	2.40E-12
CLEC3B	11.00	8.43	1.53E-16	2.40E-12
MAGEL2	11.00	8.43	1.53E-16	2.40E-12
FRAS1	11.00	8.43	1.53E-16	2.40E-12
MUC12	11.00	8.43	1.53E-16	2.40E-12
SLIT3	11.00	8.43	1.53E-16	2.40E-12
ALPK2	11.00	8.43	1.53E-16	2.40E-12
HCN4	11.00	8.43	1.53E-16	2.40E-12
IGFBP5	11.00	8.43	1.53E-16	2.40E-12
C9orf47	11.00	8.43	1.53E-16	2.40E-12
GLRA3	11.00	8.43	1.53E-16	2.40E-12
TERT	11.00	8.43	1.53E-16	2.40E-12
CLDN1	11.00	8.43	1.53E-16	2.40E-12
PLEKHS1	11.00	8.43	1.53E-16	2.40E-12
MISP	11.00	8.43	1.53E-16	2.40E-12
KSR2	11.00	8.43	1.53E-16	2.40E-12
PRSS16	11.00	8.43	1.53E-16	2.40E-12
LRRC66	11.00	8.43	1.53E-16	2.40E-12
SLC26A7	11.00	8.43	1.53E-16	2.40E-12
MUSK	11.00	8.43	1.53E-16	2.40E-12
PDYN	11.00	8.43	1.53E-16	2.40E-12
SCG2	11.00	8.43	1.53E-16	2.40E-12
MEPE	11.00	8.43	1.53E-16	2.40E-12
SLC7A13	11.00	8.43	1.53E-16	2.40E-12
ZNF474	11.00	8.43	1.53E-16	2.40E-12
SHC2	11.00	8.43	1.53E-16	2.40E-12
SUN3	11.00	8.43	1.53E-16	2.40E-12
EN2	11.00	8.43	1.53E-16	2.40E-12
ATP4B	11.00	8.43	1.53E-16	2.40E-12

BMP4	11.00	8.43	1.53E-16	2.40E-12
RASGEF1C	11.00	8.43	1.53E-16	2.40E-12
ACTL6B	11.00	8.43	1.53E-16	2.40E-12
SLC22A3	11.00	8.43	1.53E-16	2.40E-12
KLRC2	11.00	8.43	1.53E-16	2.40E-12
CXCL13	11.00	8.43	1.53E-16	2.40E-12
VCX	11.00	8.43	1.53E-16	2.40E-12
OR5K2	11.00	8.43	1.53E-16	2.40E-12
KITLG	11.00	8.43	1.53E-16	2.40E-12
TTC36	11.00	8.43	1.53E-16	2.40E-12
IFNB1	11.00	8.43	1.53E-16	2.40E-12
DPPA5	11.00	8.43	1.53E-16	2.40E-12
C6orf99	11.00	8.43	1.53E-16	2.40E-12
SLC10A6	11.00	8.43	1.53E-16	2.40E-12
LHB	11.00	8.43	1.53E-16	2.40E-12
CXCL11	11.00	8.43	1.53E-16	2.40E-12
WFDC2	11.00	8.43	1.53E-16	2.40E-12
CAPN11	11.00	8.43	1.53E-16	2.40E-12
XAGE3	11.00	8.43	1.53E-16	2.40E-12
MLN	11.00	8.43	1.53E-16	2.40E-12
IGFL3	11.00	8.43	1.53E-16	2.40E-12
SPINK1	11.00	8.43	1.53E-16	2.40E-12
HIST1H2BM	11.00	8.43	1.53E-16	2.40E-12
SLC22A31	11.00	8.43	1.53E-16	2.40E-12
DIRAS2	11.00	8.43	1.53E-16	2.40E-12
PGA3	11.00	8.43	1.53E-16	2.40E-12
FIGNL2	11.00	8.43	1.53E-16	2.40E-12
BCAR1	9.26	6.96	1.23E-11	1.93E-07
CRYGD	9.00	6.74	5.63E-11	8.83E-07
AZU1	8.96	6.70	7.17E-11	1.12E-06
NRP1	8.80	6.57	1.73E-10	2.72E-06
ELANE	8.52	6.33	7.98E-10	1.25E-05
PXMP2	8.25	6.10	3.30E-09	5.17E-05
LURAP1L	8.25	6.10	3.30E-09	5.17E-05
ABCA8	7.86	5.77	2.37E-08	3.71E-04
TPSD1	7.86	5.77	2.37E-08	3.71E-04
EDA2R	7.86	5.77	2.37E-08	3.71E-04
CABP1	7.86	5.77	2.37E-08	3.71E-04
GPR15	7.75	5.68	3.97E-08	6.23E-04
BUB1B	7.40	5.39	2.01E-07	3.14E-03
PTCRA	7.33	5.33	2.76E-07	4.33E-03
C2orf80	7.33	5.33	2.76E-07	4.33E-03
MARCH2	7.33	5.33	2.76E-07	4.33E-03
NDNF	7.33	5.33	2.76E-07	4.33E-03
STEAP1B	7.33	5.33	2.76E-07	4.33E-03
OR52I2	7.33	5.33	2.76E-07	4.33E-03
GRK1	7.33	5.33	2.76E-07	4.33E-03
SMIM10	6.88	4.94	2.02E-06	3.16E-02
CTSG	6.87	4.93	2.08E-06	3.26E-02

Table S7: Primer sequences

Target	Primer ID	Sequence (5' to 3')	Annealing T (°C)
MPO Exon 1	MPO Ex1 F	CTTCCTCTACCTCACCCCAC	62
	MPO Ex1 R	CTATCAGGCCCCAGAGCTAG	
MPO Exon 2	MPO Ex2 F	TTCCTAGCTCTGGGGCCT	62
	MPO Ex2 R	CCTCTCCCACCTTCAAGCT	
MPO Exon 3	MPO Ex3 F	CAAAGCCTTGCCTCTGTCTG	62
	MPO Ex3 R	TGGAGGAAGAAGTTGAGGGG	
MPO Exon 4-5	MPO Ex4-5 F	CCCCTCAACTTCTTCCTCCA	62
	MPO Ex4-5 R	TCAGCTGATCAGTGGGGAAG	
MPO Exon 6	MPO Ex6 F	GCCAGCTGATCTCCGTGT	62
	MPO Ex6 R	CAGCGTCTGGGAAAGGAAAC	
MPO Exon 7	MPO Ex7 F	CTGCTCATTAACCCTGCACC	62
	MPO Ex7 R	CCACAAGCTGCTCACAAACA	
MPO Exon 8	MPO Ex8 F	GGGGTTTCAGTGGAGCAAAT	62
	MPO Ex8 R	TCAACCCTCCCAACACCAAT	
MPO Exon 9	MPO Ex9 F	CCAAGAGCAGGCAGAGACT	64
	MPO Ex9 R	AGGCTAGAGAGTCAGACCAGA	
MPO Exon 10	MPO Ex10 F	TCTCGAATCCTCCTGACCCT	64
	MPO Ex10 R	TCTAATATGCTTTGGAGAGGGC	
MPO Exon 11	MPO Ex11 F	TCTCCAGTGACCTCCCCA	62
	MPO Ex11 R	AGGAGGAAATTTGGGCTCCA	
MPO Exon 12	MPO Ex12 F	ATATCCTGGGAGCAGCACAA	62
	MPO Ex12 R	CATTTTCTCAGCTGCACCCA	

Supplemental references

1. Navarini AA, Burden AD, Capon F, Mrowietz U, Puig L, Koks S, et al. European Consensus Statement on Phenotypes of Pustular Psoriasis. J Eur Acad Dermatol Venereol 2017:1792-9.
2. Mahil SK, Twelves S, Farkas K, Setta-Kaffetzi N, Burden AD, Gach JE, et al. AP1S3 Mutations Cause Skin Autoinflammation by Disrupting Keratinocyte Autophagy and Up-Regulating IL-36 Production. J Invest Dermatol 2016:2251-9.
3. Sidoroff A, Halevy S, Bavinck JN, Vaillant L, Roujeau JC. Acute generalized exanthematous pustulosis (AGEP)--a clinical reaction pattern. J Cutan Pathol 2001; 28:113-9.
4. Onoufriadis A, Simpson MA, Pink AE, Di Meglio P, Smith CH, Pullabhatla V, et al. Mutations in IL36RN/IL1F5 are associated with the severe episodic inflammatory skin disease known as generalized pustular psoriasis. Am J Hum Genet 2011; 89:432-7.
5. Setta-Kaffetzi N, Navarini AA, Patel VM, Pullabhatla V, Pink AE, Choon SE, et al. Rare Pathogenic Variants in IL36RN Underlie a Spectrum of Psoriasis-Associated Pustular Phenotypes. J Invest Dermatol 2013; 133:1366-9.
6. Li H, Handsaker B, Wysoker A, Fennell T, Ruan J, Homer N, et al. The Sequence Alignment/Map format and SAMtools. Bioinformatics 2009; 25:2078-9.
7. Wang K, Li M, Hakonarson H. ANNOVAR: functional annotation of genetic variants from high-throughput sequencing data. Nucleic Acids Res 2010; 38:e164.
8. Ruark E, Munz M, Renwick A, Clarke M, Ramsay E, Hanks S, et al. The ICR1000 UK exome series: a resource of gene variation in an outbred population. F1000Res 2015; 4:883.
9. Kircher M, Witten DM, Jain P, O'Roak BJ, Cooper GM, Shendure J. A general framework for estimating the relative pathogenicity of human genetic variants. Nat Genet 2014; 46:310-5.
10. Catapano M, Vergnano M, Romano M, Mahil SK, Choon SE, Burden AD, et al. Interleukin-36 promotes systemic Type-I IFN responses in severe psoriasis. Journal of Investigative Dermatology 2019; in press.
11. Canela-Xandri O, Rawlik K, Tenesa A. An atlas of genetic associations in UK Biobank. Nat Genet 2018; 50:1593-9.
12. Karczewski KJ, Francioli LC, Tiao G, Cummings BB, Alfoldi J, Wang Q, et al. Variation across 141,456 human exomes and genomes reveals the spectrum of loss-of-function intolerance across human protein-coding genes. BioRxiv 2019.
13. Stendahl O, Lindgren S. Function of granulocytes with deficient myeloperoxidase-mediated iodination in a patient with generalized pustular psoriasis. Scand J Haematol 1976; 16:144-53.

14. De Argila D, Dominguez JD, Lopez-Estebaranz JL, Iglesias L. Pustular psoriasis in a patient with myeloperoxidase deficiency. Dermatology 1996; 193:270.

15. Nguyen C, Katner HP. Myeloperoxidase deficiency manifesting as pustular candidal dermatitis. Clin Infect Dis 1997; 24:258-60.

16. Disdier P, Harle JR, Weiller-Merli C, Andrac L, Weiller PJ. Neutrophilic dermatosis despite myeloperoxidase deficiency. J Am Acad Dermatol 1991; 24:654-5.
